# Chromatin activation as a unifying principle underlying pathogenic mechanisms in multiple myeloma

**DOI:** 10.1101/gr.265520.120

**Published:** 2020-09

**Authors:** Raquel Ordoñez, Marta Kulis, Nuria Russiñol, Vicente Chapaprieta, Arantxa Carrasco-Leon, Beatriz García-Torre, Stella Charalampopoulou, Guillem Clot, Renée Beekman, Cem Meydan, Martí Duran-Ferrer, Núria Verdaguer-Dot, Roser Vilarrasa-Blasi, Paula Soler-Vila, Leire Garate, Estíbaliz Miranda, Edurne San José-Enériz, Juan R. Rodriguez-Madoz, Teresa Ezponda, Rebeca Martínez-Turrilas, Amaia Vilas-Zornoza, David Lara-Astiaso, Daphné Dupéré-Richer, Joost H.A. Martens, Halima El-Omri, Ruba Y. Taha, Maria J. Calasanz, Bruno Paiva, Jesus San Miguel, Paul Flicek, Ivo Gut, Ari Melnick, Constantine S. Mitsiades, Jonathan D. Licht, Elias Campo, Hendrik G. Stunnenberg, Xabier Agirre, Felipe Prosper, Jose I. Martin-Subero

**Affiliations:** 1 Centro de Investigación Médica Aplicada (CIMA), IDISNA, 31008 Pamplona, Spain;; 2 Centro de Investigación Biomédica en Red de Cáncer, CIBERONC, 28029 Madrid, Spain;; 3 Fundació Clínic per a la Recerca Biomèdica, 08036 Barcelona, Spain;; 4 Institut d'Investigacions Biomèdiques August Pi I Sunyer (IDIBAPS), 08036 Barcelona, Spain;; 5 Departamento de Fundamentos Clínicos, Universitat de Barcelona, 08036 Barcelona, Spain;; 6 Division of Hematology/Oncology, Department of Medicine, Weill Cornell Medical College, New York, New York 10021, USA;; 7 Clínica Universidad de Navarra, 31008 Pamplona, Spain;; 8 Division of Hematology/Oncology, University of Florida Health Cancer Center, Gainesville, Florida 32610, USA;; 9 Radboud Institute for Molecular Life Sciences, 6525 GA Nijmegen, Netherlands;; 10 Department of Hematology & BMT, Hamad Medical Corporation, NCCCR, Doha, Qatar;; 11 European Molecular Biology Laboratory, European Bioinformatics Institute (EMBL-EBI), Wellcome Trust Genome Campus, Hinxton CB10 1SD, United Kingdom;; 12 CNAG-CRG, Centre for Genomic Regulation (CRG), Barcelona Institute of Science and Technology (BIST), 08028 Barcelona, Spain;; 13 Department of Medical Oncology, Dana-Farber Cancer Institute, Harvard Medical School, Boston, Massachusetts 02215, USA;; 14 Institució Catalana de Recerca i Estudis Avançats (ICREA), 08010 Barcelona, Spain

## Abstract

Multiple myeloma (MM) is a plasma cell neoplasm associated with a broad variety of genetic lesions. In spite of this genetic heterogeneity, MMs share a characteristic malignant phenotype whose underlying molecular basis remains poorly characterized. In the present study, we examined plasma cells from MM using a multi-epigenomics approach and demonstrated that, when compared to normal B cells, malignant plasma cells showed an extensive activation of regulatory elements, in part affecting coregulated adjacent genes. Among target genes up-regulated by this process, we found members of the NOTCH, NF-kB, MTOR signaling, and TP53 signaling pathways. Other activated genes included sets involved in osteoblast differentiation and response to oxidative stress, all of which have been shown to be associated with the MM phenotype and clinical behavior. We functionally characterized MM-specific active distant enhancers controlling the expression of thioredoxin (*TXN*), a major regulator of cellular redox status and, in addition, identified *PRDM5* as a novel essential gene for MM. Collectively, our data indicate that aberrant chromatin activation is a unifying feature underlying the malignant plasma cell phenotype.

Multiple myeloma (MM) is an aggressive hematological neoplasm characterized by the uncontrolled expansion and accumulation of malignant plasma cells (PCs) in the bone marrow (San Miguel 2014; Kumar et al. 2017). Given the central role of genomic alterations in cancer development, the study of molecular mechanisms underlying MM pathogenesis has mostly focused on the genetic aberrations of these tumors (Stratton et al. 2009). Such studies have revealed that MM patients are genetically heterogeneous, without a single and unifying genetic event identified in all patients (Robiou du Pont et al. 2017), but nevertheless the central paradox of MM remains, that is, terminally differentiated plasma cells normally do not divide. This indicates that the essential aspects of the gene regulatory networks within the malignant plasma cell are dysfunctional and suggests that epigenetic deregulation of gene expression may be a root cause of the disease. Over the last decades, a greater understanding of the role of histone, DNA, and other chromatin modifications in the epigenetic control of gene expression has transformed our understanding of transcriptional patterns in normal and neoplastic cells (Allis and Jenuwein 2016). In spite of the multifaceted nature of the epigenome (Stricker et al. 2017), the cancer epigenomics field has been mostly focused on the association between DNA methylation and transcription (Esteller 2008). However, recent whole-genome DNA methylation studies indicate that this association is less clear than previously appreciated (Kulis et al. 2012, 2015; Kretzmer et al. 2015) and that understanding gene deregulation in cancer requires the integrative analysis of various epigenetic marks including histone modifications and chromatin accessibility (Martens et al. 2010; Ooi et al. 2016; Beekman et al. 2018). In MM, although several reports have identified alterations in the DNA methylome of malignant plasma cells (Walker et al. 2011; Heuck et al. 2013; Kaiser et al. 2013; Agirre et al. 2015), their pathogenic impact has been uncertain and the chromatin regulatory network underlying aberrant cellular functions in MM has just started to be characterized (Agarwal et al. 2016; Jin et al. 2018).

## Results

### Integrative analysis of multiple epigenetic layers in MM

**Figure 1.**
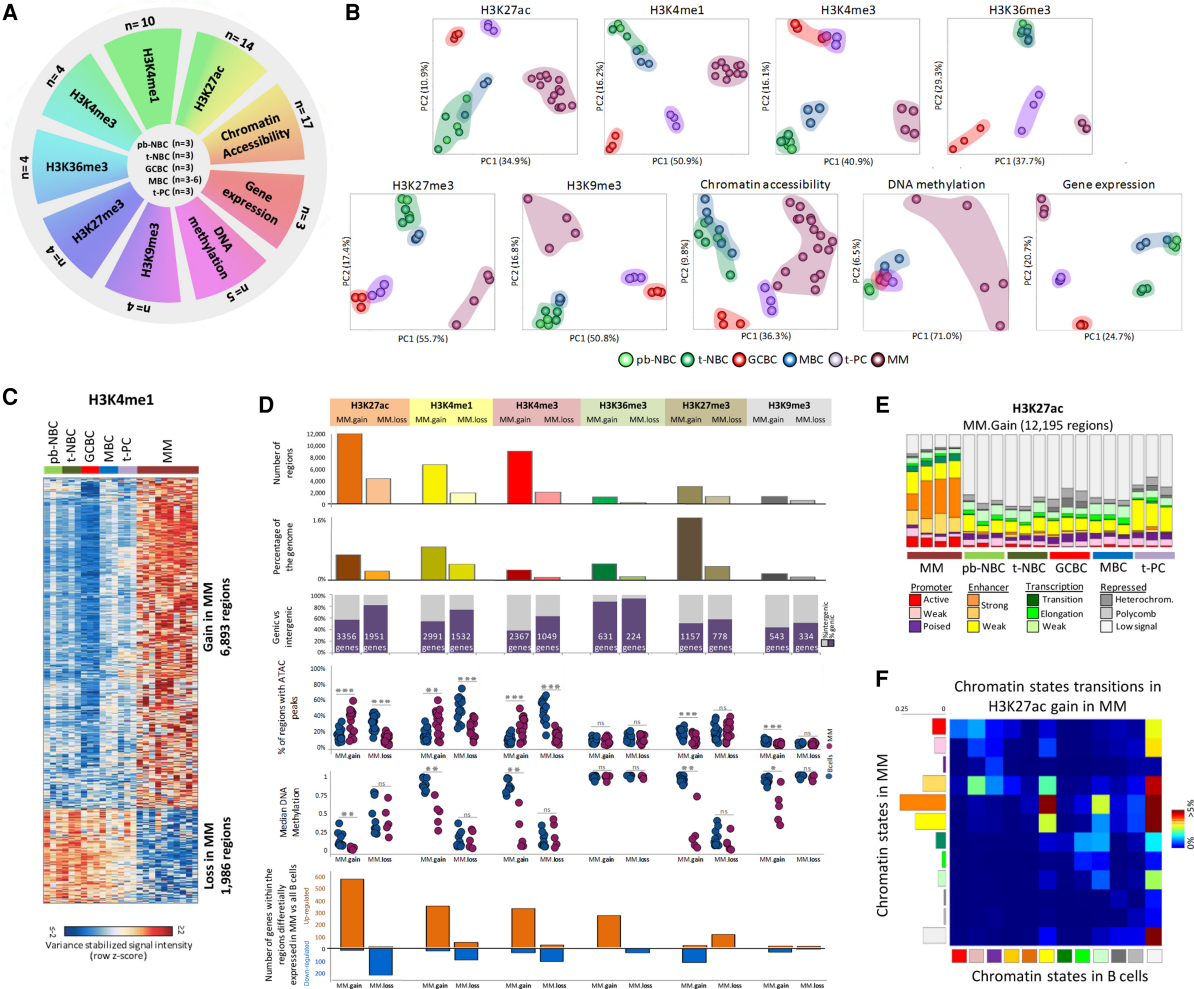
Initial characterization of epigenetic layers in multiple myeloma. (*A*) Schematic representation of the experimental design. The outer circle shows the numbers of MM samples for the nine epigenetic layers used in the study, while numbers of normal B cell samples are shown in the center. (*B*) Unsupervised principal component analysis for the nine layers of the epigenome. (*C*) Heat map representation of the regions with differential H3K4me1 occupancy in MM as compared to a stable pattern throughout normal B cell differentiation. (*D*) Characterization of regions with stable chromatin profiles throughout B cell differentiation showing either gain or loss of a specific histone mark in MM. From *upper* to *lower* panel: barplot showing number of regions detected for each condition; barplot showing total occupancy of the differential histone mark regions as a percentage of the whole genome; fractions of regions located in intergenic regions or inside genes (number of host genes associated with the differential regions shown in the graph); fraction of regions in MM (*n* = 17) and normal B cells (*n* = 15) harboring ATAC-seq peaks within regions with increase or decrease of particular histone marks in MM; median DNA methylation levels in MM (*n* = 5) and normal B cells (*n* = 12) within the regions with increase or decrease of particular histone mark in MM; barplots presenting number of host genes associated with the differential histone mark regions that are up-regulated or down-regulated in MM as compared to normal B cells (FDR < 0.05; |FC|>1.5). (*E*) Distribution of the different chromatin states in all analyzed samples separately at regions with increase of H3K27ac in MM as compared to normal B cells. (*F*) Chromatin state transition matrix for regions with increase of H3K27ac in MM as compared to normal B cells. Columns represent the chromatin state in normal B cells and rows are chromatin states in MMs that arise from normal B cells. The total matrix represents 100% of the differential regions. All *P*-values were calculated using a Student's *t*-test (two-sided). (*) *P* < 0.05, (**) *P* < 0.01, (***) *P* < 0.001, (ns) not significant. (pb-NBC) Naive B cells from blood, (t-NBC) naive B cells from tonsils, (GCBC) germinal center B cells, (MBC) memory B cells, (t-PC) plasma cell from tonsils, (MM) multiple myeloma.

We performed an integrative analysis of multiple epigenetic layers in purified plasma cells from three MM patients, including whole-genome maps of six core histone modifications (i.e., H3K27ac, H3K4me1, H3K4me3, H3K36me3, H3K27me3, and H3K9me3) by ChIP-seq, chromatin accessibility by ATAC-seq, DNA methylation by whole-genome bisulfite sequencing (WGBS), and gene expression by RNA-seq. The major limiting factor for such multi-omics characterization was the high amount of starting material needed from each individual patient. To overcome this limitation, we also analyzed the chromatin regulatory landscape of an extended series of MM patients, including additional profiles for H3K27ac (*n* = 11), H3K4me1 (*n* = 7), ATAC-seq (*n* = 14), and RNA-seq data (*n* = 37). Normal controls consisted of reference epigenomes from B cell subpopulations at different maturation stages, including naive B cells (both from peripheral blood, pb-NBCs, and tonsils, t-NBCs), germinal center B cells (GCBCs), memory B cells (MBCs), and tonsillar PCs (t-PCs) (Fig. 1A; Supplemental Tables S1, S2; Beekman et al. 2018). Additionally, we were able to characterize the H3K27ac and transcriptional profiles of bone marrow plasma cells (bm-PCs), the normal counterpart of MM, from which a complete reference epigenome could not be generated due to the scarcity of this cell subpopulation in healthy bone marrow.

Unsupervised principal component analysis indicated that MM displays an epigenetic configuration distinct from normal B cell subpopulations, which is reflected in every single layer of the epigenome (Fig. 1B). As B cell differentiation entails modulation of all studied epigenetic layers, we focused our analysis on the genome fraction changing in MM but showing stable chromatin profiles across the normal B cell maturation program. This strategy will allow us to identify chromatin changes specifically altered during myelomagenesis (Fig. 1C; Supplemental Figs. S1, S2). Overall, we observed that each histone modification undergoes more gains than losses in MM but at different degrees, with the H3K27ac, H3K4me1, and H3K4me3 regulatory marks being those with the largest number of gained regions (Fig. 1D), even when normalized by the total number of peaks detected by each mark (Supplemental Fig. S3). This finding points to a gain of regulatory elements such as enhancers and promoters in MM (Fig. 1E; Supplemental Fig. S4) that frequently arise from low-signal heterochromatic regions in normal B cells (Fig. 1F; Supplemental Fig. S5). These regions were associated with more accessible chromatin, loss of DNA methylation, and an increased expression of the associated genes in MM as compared to normal B cells (Fig. 1D). Loss of regulatory elements in MM was related to the opposite patterns, with the exception of DNA methylation, which showed similar levels in normal B cells and MM in these regions. This finding indicates that once a region has become active and demethylated earlier in B cell differentiation, this methylation state is maintained regardless of changes in activity, thus retaining a memory of past activation, as previously described (Kulis et al. 2015; Beekman et al. 2018). Myelomagenesis-related changes in H3K36me3, a transcriptional elongation mark, were associated with gene expression changes in MM in the absence of chromatin accessibility or DNA methylation alterations. These regions were highly DNA-methylated both in normal and neoplastic cells regardless of the modulation of H3K36me3 levels, and we ruled out any significant modulation of DNA hydroxymethylation levels (Supplemental Fig. S6). Such high DNA methylation in elongating gene bodies has been associated with recruitment of DNA methyltransferases DNMT3A/B by H3K36me3 (Neri et al. 2017; Teissandier and Bourc'his 2017). MM-specific changes in H3K27me3 and H3K9me3 showed minor or absent modulation of chromatin accessibility and gene expression levels. In contrast, regions gaining these repressive marks in MM showed a marked loss of DNA methylation, which is counterintuitive, as these chromatin marks are conventionally known to be associated with methylated DNA (Viré et al. 2006; Schlesinger et al. 2007; Brinkman et al. 2012). We analyzed the DNA methylome of our MM samples in further detail and observed extensive DNA hypomethylation taking place in inactive chromatin regions regardless of the genomic location (within or outside gene bodies) and the particular mode of repression, that is, presence of H3K27me3, presence of H3K9me3, or low signal heterochromatin (Fig. 1D; Supplemental Figs. 7A,B, S8). Additionally, we could identify that regions changing H3K27me3 or H3K9me3 frequently reflect transitions among repressive chromatin states from normal to neoplastic cells (Supplemental Fig. S5), and DNA methylation in MM seems to decrease in repressed chromatin independently of these transitions (Supplemental Fig. S7C). This phenomenon of DNA hypomethylation in repressed, late-replicating regions has been recently linked to mitotic cell division rather than to a regulatory function (Zhou et al. 2018). Collectively, these findings indicate that MM is characterized by a highly dynamic chromatin landscape, which, on the one hand, affects heterochromatin without apparent functional impact and, on the other hand, leads to an emergence of active regulatory elements leading to extensive perturbation of the MM transcriptome.

### De novo chromatin activation affects genes related to key pathogenic mechanisms in MM

**Figure 2.**
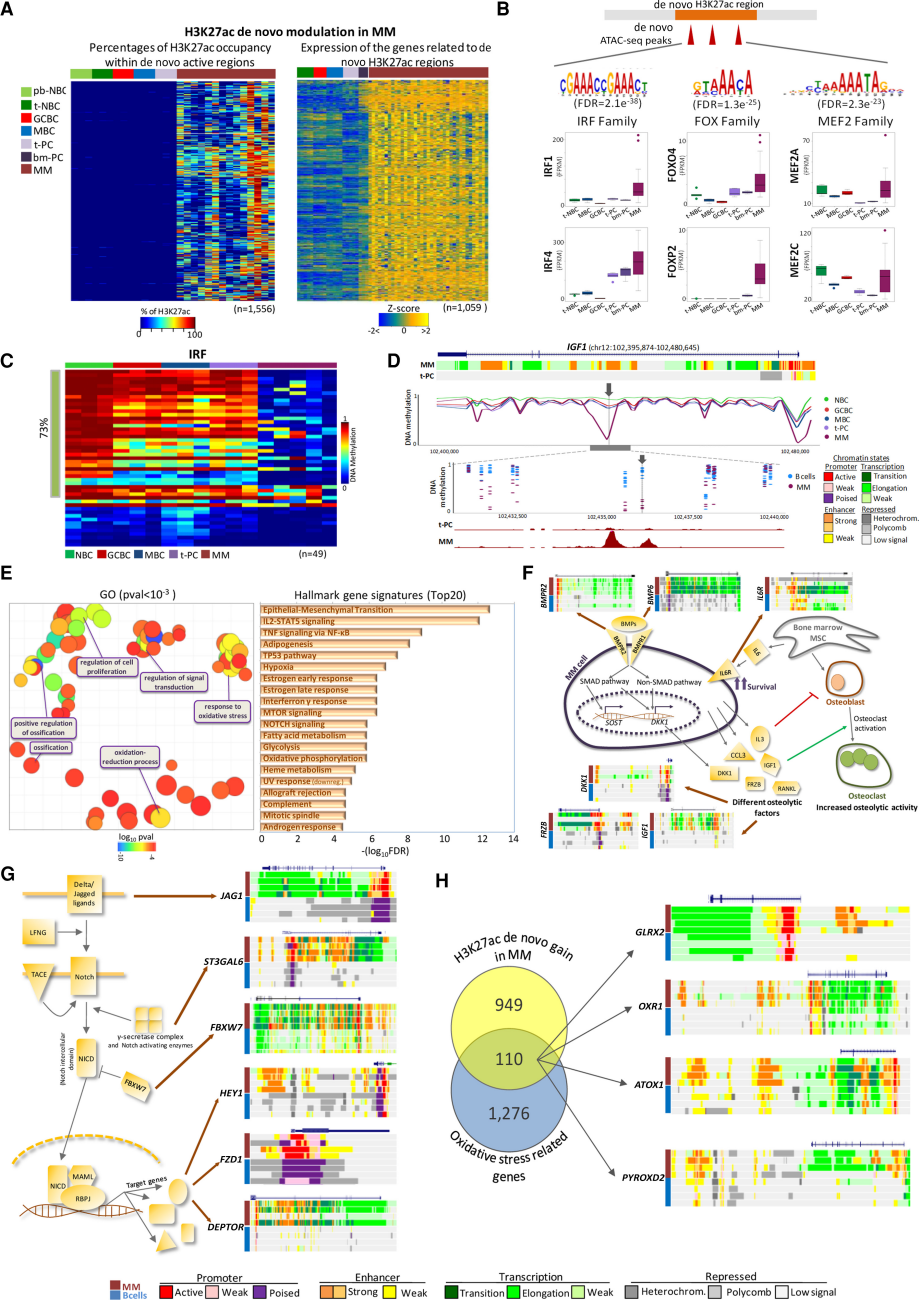
Functional impact of de novo chromatin activation in MM. (*A*) Heat maps representing the percentage of the regions covered by H3K27ac peak within the de novo activated regions in MM (*left* panel) and expression of the genes associated with these regions. (*B*) TF families motifs enriched in the de novo activated regions in MM, that is, IRF, FOX, and MEF2, as identified by MEME analysis. For each TF family, expression of two selected members up-regulated in MM as compared to normal B cells is shown. (*C*) Heat map representing methylation levels of all CpGs (*n* = 49) identified within IRF motifs, in different subpopulations of normal B cells and MM samples. Green bar at *left* marks the CpGs hypomethylated in MM. (*D*) Example of DNA methylation profiles within *IGF1* gene. *Upper* panel shows a global view of a whole gene, with chromatin state track of MM and t-PC, while the *lower* panels represent the zoom to the IRF motif locus. Gray arrow shows CpG within IRF motif. (*E*) Gene Ontology results, shown as semantic-similarity scatterplot of the most significant GO terms (*P* < 0.001), summarized together by REVIGO software (*left* panel) and a list of top 20 hallmark gene signatures, determined using MSigDB Collection (*right* panel). (*F*) Schematic representation of mechanisms involved in interactions between MM and the bone marrow microenvironment, with selected genes harboring activated chromatin in MM as compared to normal B cells. (*G*) Schematic representation of the NOTCH signaling pathway with selected genes harboring activated chromatin in MM as compared to normal B cells. (*H*) Venn diagram presenting the overlap of genes associated with de novo active regions in MM and genes belonging to GOs related with oxidative stress (i.e., oxidative-reduction process and response to oxidative stress). Chromatin states within selected genes in MM and normal B cells are shown on the *right* panel. (bm-PC) Plasma cell from bone marrow.

To detect regulatory elements that could represent MM-specific epigenetic alterations, we aimed at identifying de novo active regions by focusing on H3K27ac, a bona fide indicator of enhancer and promoter activation (Zentner et al. 2011; Bonn et al. 2012). In this way, we could select regions without any H3K27ac peak across normal B cell differentiation (i.e., inactive) while gaining this histone mark specifically in MM. Although due to the scarcity of plasma cells in the healthy bone marrow, it was not feasible to generate multiple epigenetic marks in this cellular subpopulation, we included bm-PC in this analysis as a key filter for H3K27ac and RNA-seq. We know that this information is limited, but it has been very important in our study to discard changes associated with cell of origin (Supplemental Fig. S9). Using this strategy, we retained 1556 regions, which were used for further downstream analyses (Supplemental Table S3). From these individual regions, 13.5% (*n* = 210) corresponded to 115 predicted superenhancers (Supplemental Table S4). Then, using a combined approach that considers topologically associating domains (TADs) from the mature B cell line GM12878 and transcript levels from MM cells and normal B cells (see the Methods section for further details), these 1556 regions were associated with a total of 1059 target genes (Fig. 2A; Supplemental Table S3). Within these 1556 de novo active regions, we identified 806 sites that also increased chromatin accessibility in MM as compared to normal B cells. These sites were enriched in binding motifs of *IRF*, *FOX*, and *MEF2* transcription factor (TF) families (Fig. 2B; Supplemental Table S5), which have been previously linked to MM pathogenesis (Carvajal-Vergara et al. 2005; Shaffer et al. 2008; Campbell et al. 2010; Bai et al. 2017; Agnarelli et al. 2018). As binding of TFs is associated with local DNA demethylation (Kulis et al. 2013; Ziller et al. 2013; Sardina et al. 2018), we studied the methylation levels of CpGs located within or close to the enriched TF binding motifs. We observed that the majority of these CpGs were hypomethylated in MM as compared to normal B cells (57%–74% of CpGs with methylation difference >0.25) (Fig. 2C; Supplemental Fig. S10) and that DNA methylation levels sharply decreased in TF binding regions as compared to surrounding areas (Fig. 2D; Supplemental Fig. S11). In addition, we observed that some members of these three TF families are overexpressed in MM as compared to normal PCs, such as *IRF1*, *IRF4*, *FOXO4*, *FOXP2*, *MEF2A*, and *MEF2C* (Fig. 2B; Supplemental Table S6). Whereas part of them are de novo expressed in MM as compared to normal B cell differentiation, that is, *IRF1* and *FOXP2*, others show elevated levels in normal PCs and are further up-regulated in MM, that is, *IRF4* and *FOXO4*, and a third group is down-regulated in normal PCs as compared to NBCs, GCBs, and MBCs but showed increased expression in MM (Fig. 2B). These results support the hypothesis that extensive chromatin activation in MM may not only be mediated by disease-specific overexpression of TFs but also by exploiting and enhancing the function of TFs modulated during normal B cell differentiation.

**Figure 3.**
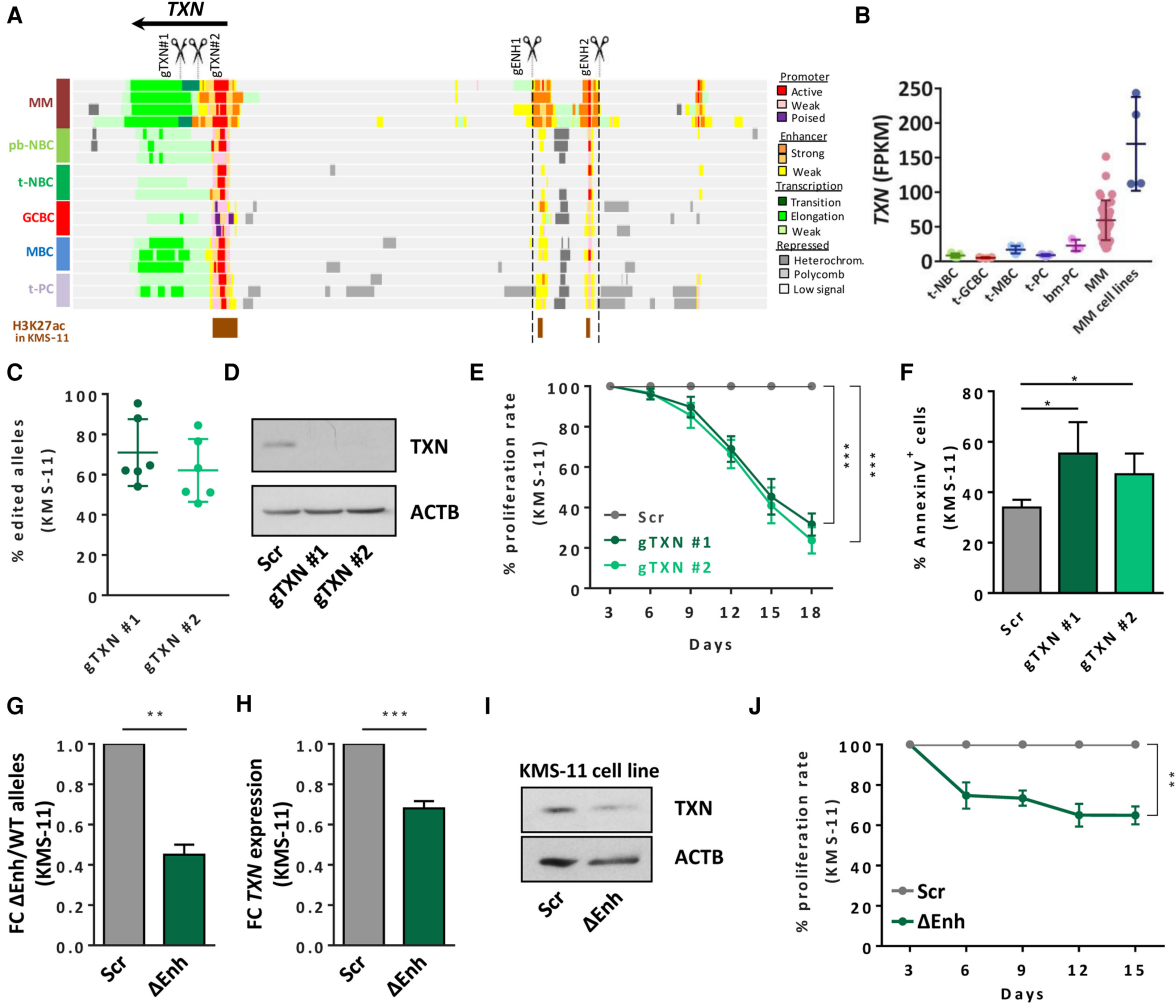
*TXN* de novo activated enhancer as an essential regulatory element in MM. (*A*) Genome browser snapshot of the *TXN* locus and the associated enhancer de novo activated in MM located at 50 kb from the promoter region. Displayed tracks represent the chromatin state annotation in MM patients and normal B cells and an additional track of H3K27ac peaks in KMS-11 cell line (ENCODE Consortium, ENCSR715JBO). gRNA design strategy for TXN knockout and enhancer deletion is also shown. (*B*) *TXN* expression in MM patients and cell lines analyzed by RNA-seq. (*C*) Estimation of the allelic cell population percentage exhibiting indel events in the targeted site, analyzed by Tracking of Indels by DEcomposition (TIDE) web tool. (*D*) Validation of *TXN* reduced expression by western blot analysis in KMS-11 cell line. (*E*) Cell proliferation assay comparing the growth proliferation rates of scramble cells (Scr) and cells harboring two different gRNAs, as determined by flow cytometry analysis. (*F*) Effect of *TXN* reduced expression in cell apoptosis, as determined by Annexin V flow cytometry analysis. (*G*) Quantification of *TXN* enhancer deletion by genomic DNA qPCR normalized to a distal nontargeted genomic region, represented as fold change of deleted enhancer (ΔEnh) versus wild-type (WT) alleles. (*H*) *TXN* mRNA expression levels determined by RT-qPCR. (*I*) TXN protein levels in cells with deleted enhancer region (ΔEnh) and scrambled cells (Scr) determined by western blot. (*J*) Cell proliferation assay comparing the growth proliferation rates of scramble cells and cells harboring the enhancer deletion (ΔEnh), as determined by flow cytometry analysis. (*) *P* < 0.05, (**) *P* < 0.01, (***) *P* < 0.001.

Next, to decipher the downstream pathogenic relevance of de novo active regulatory regions in MM, we explored the functional categories associated with the target genes (Fig. 2E; Supplemental Table S7). The results point to a variety of functions previously described to be altered in MM (Bommert et al. 2006; Hu and Hu 2018), including regulation of osteoblast differentiation, multiple signaling pathways, such as NF-kB signaling, MTOR signaling, the TP53 pathway, and the NOTCH pathway, as well as oxidative stress responses (Fig. 2E). Figure 2, F through H, shows examples of genes with regulatory elements becoming de novo active in MM as compared to normal cells. For instance, in the case of the NOTCH pathway, we identified chromatin activation of genes at different steps of the pathway, starting from de novo active ligands, receptor processing machinery, up to downstream targets (Fig. 2G). As activation of the above-mentioned pathways is in part mediated by the crosstalk between MM and microenvironmental cells, our results suggest that such microenvironmental interactions may underlie chromatin activation of downstream genes in MM. Additionally, we observed a significant association with GO terms and hallmark signatures involved in oxidative stress responses (Fig. 2H). In fact, oncogenic transformation in MM is accompanied by higher endoplasmic reticulum and oxidative stresses mostly because of high immunoglobulin production and increased metabolic demands caused by the proliferative activity of MM cells (White-Gilbertson et al. 2013; Lipchick et al. 2016). Our findings suggest that MM cells activate a specific regulatory network to be able to survive in spite of elevated oxidative stress conditions. This network seems to be related to the emergence of de novo enhancers leading to overexpression of genes involved in major detoxification systems, including, for example, *GLRX*, *OXR1*, *ATOX1*, *PYROXD2*, and thioredoxin (*TXN*) (Figs. 2H, 3A).

### *TXN* de novo activated enhancer is an essential regulatory element in MM

TXN contributes to maintain reactive oxygen species (ROS) homeostasis in the cell to prevent oxidative damage (Arnér and Holmgren 2006) and has been recently shown to enhance cell growth in MM, and its inhibition leads to ROS-induced apoptosis in MM cell lines (Raninga et al. 2015; Sebastian et al. 2017). From the chromatin perspective, the *TXN* gene body and promoter show activating histone modifications both in MM and normal B cell subpopulations. Furthermore, we identified a de novo active distant enhancer region, located ∼50 kb upstream of the *TXN* transcription start site (TSS) (Fig. 3A). Using GM12878 as a cellular model, Hi-C data (GSE63525) (Rao et al. 2014) revealed that the active enhancer elements showed significant 3D interactions to the *TXN* promoter region. The presence of this loop and the fact that *TXN* is the only overexpressed gene within the TAD strongly suggest that *TXN* is the only target gene of the identified enhancer elements (Supplemental Fig. S11). This overall active chromatin was associated with *TXN* overexpression in both MM patients and cell lines compared to normal B cell differentiation (Fig. 3B; Supplemental Fig. S12A). We further aimed to characterize the pathogenic implication of *TXN* deregulation in MM cells. *TXN* inactivation using the CRISPR-Cas9 strategy in MM cell lines (Fig. 3C,D; Supplemental Figs. S12B,C, S13) led to a significant reduction of growth rate as compared to control cells (Fig. 3E; Supplemental Fig. S12D). Such slower proliferation rate is associated with an increased apoptotic phenotype, as measured by Annexin V flow cytometry analysis (Fig. 3F; Supplemental Fig. S12E). Additionally, we observed a trend toward higher ROS production after *TXN* silencing (Supplemental Fig. S14), consistent with previous reports (Raninga et al. 2015; Sebastian et al. 2017). These results corroborate that *TXN* is an essential gene for MM cell survival and proliferation (Raninga et al. 2015, 2016; Zheng et al. 2018). We then studied whether *TXN* overexpression was driven by the de novo activation of the distant enhancer identified in MM patients. We designed a CRISPR-Cas9 paired gRNAs system, with two plasmids carrying gRNAs flanking the regulatory region, to completely delete the 11-kb *TXN* enhancer identified in MM patients (Fig. 3A). We observed that enhancer deletion in bulk cells significantly reduced *TXN* expression at the RNA and protein level in a MM cell line and was associated with a significant decrease in cell proliferation (Fig. 3G–J; Supplemental Fig. S15). Taken together, our results extend previous reports on the essential role of *TXN* in MM and show that de novo activation of a distant enhancer underlies its overexpression and pathogenic impact.

### *PRDM5* as a new candidate oncogene in MM

**Figure 4.**
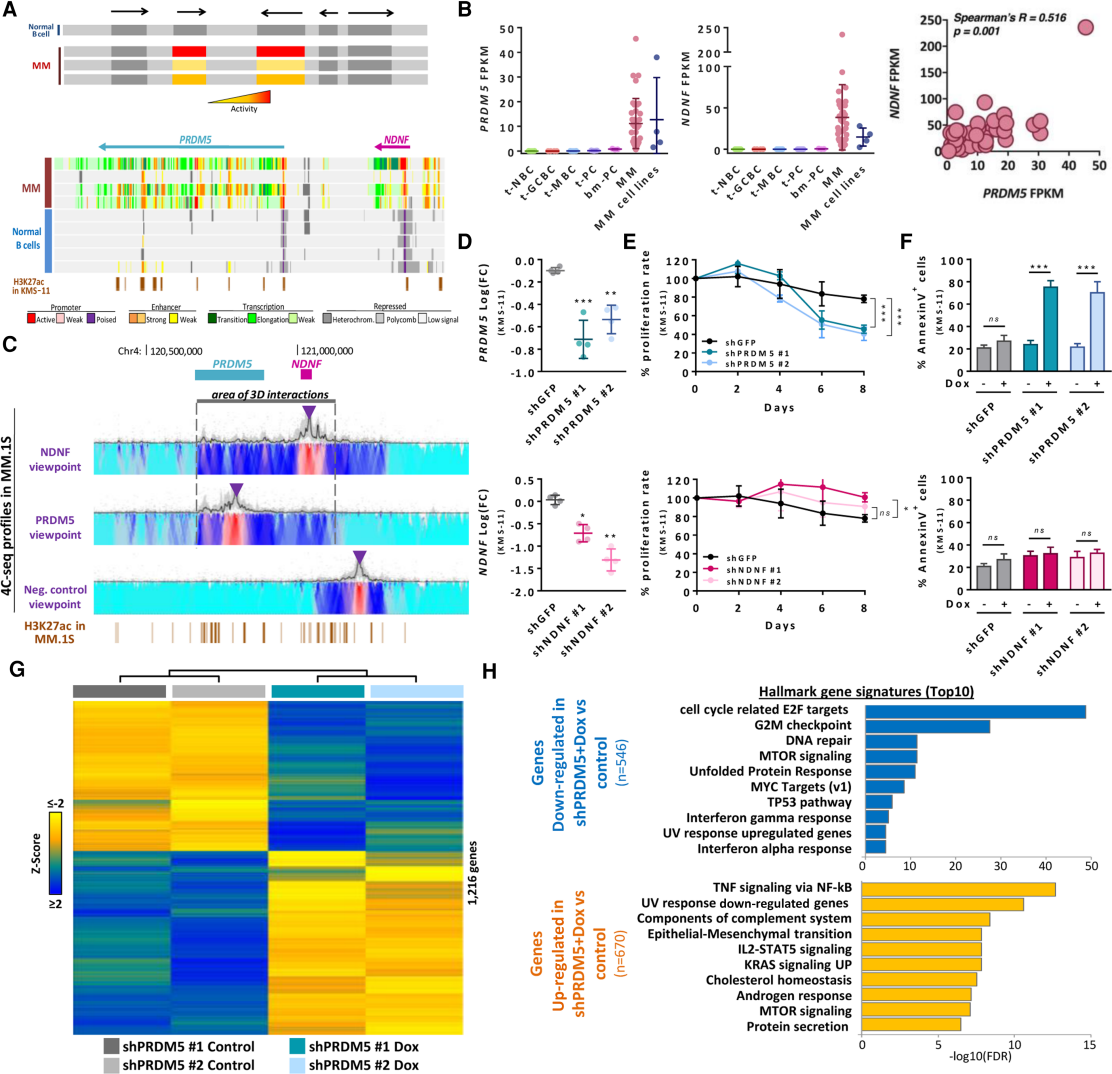
Identification of *PRDM5* as a new candidate oncogene in MM. (*A*) Schematic strategy for identification of coregulated chromatin regions. Genome browser snapshot of the coregulated chromatin region comprising the *PRDM5* and *NDNF* locus. Displayed tracks represent the chromatin state annotation in MM patients and normal B cells, and an additional track of H3K27ac peaks in KMS-11 cell line (ENCODE Consortium, ENCSR715JBO). (*B*) *PRDM5* and *NDNF* expression analyzed by RNA-seq (*left* and *center* panel), and correlated expression levels in MM patients (*right* panel). (*C*) Normalized levels of chromatin interaction frequencies from the indicated viewpoint (purple arrowhead) between *PRDM5* and *NDNF* gene loci as analyzed by 4C-seq in the MM.1S MM cell line expressing both transcripts. Lowest contact frequencies are indicated in turquoise, and highest frequencies in red. *PRDM5* and *NDNF* location are shown above the 4C-seq track; black bar represents area of increased interactions, while H3K27ac peaks in this cell line are shown below. (*D*) Validation of *PRDM5* (*upper* panel) and *NDNF* (*lower* panel) knockdown after shRNA expression determined by RT-qPCR. Expression values are normalized to the same condition prior to addition of doxycycline. Statistical analysis compares the effect of each shRNA versus the scramble shRNA (shGFP). (*E*) Relative cell proliferation rate (%) of KMS-11 cell line normalized to the same condition prior to addition of doxycycline. The *upper* panel presents the results for *PRDM5* knockdown cells and the *lower* panel for *NDNF* knockdown cells. Statistical analysis compares the effect of each shRNA versus the scramble shRNA (shGFP). (*F*) Effect of *PRDM5* (*upper* panel) and *NDNF* (*lower* panel) knockdown in cell apoptosis, as determined by Annexin V flow cytometry analysis. (*G*) Heat map of gene expression levels (indicated as *Z*-scores) of differentially expressed genes in *PRDM5* knockdown cells before and after 4 d of the addition of doxycycline (*n* = 2 for control and Dox groups; 1216 genes). (*H*) List of top 10 hallmark gene signatures, determined using MSigDB Collection for genes down-regulated (*upper* panel) or up-regulated (*lower* panel) in *PRDM5* knockdown cells 4 d after the addition of doxycycline. (Dox) Doxycycline. (*) *P* < 0.05, (**) *P* < 0.01, (***) *P* < 0.001, (ns) not significant.

Beyond the activation of genes affecting relevant pathogenic mechanisms in MM, we observed that adjacent genes without evident functional association become de novo active in MM. This suggests the presence of coregulated chromatin regions that can coordinate the deregulation of more than one target gene specifically in tumor plasma cells (Fig. 4A). To capture these coregulated chromatin regions, we selected adjacent coexpressed genes (Pearson's R > 0.5, *P* < 0.05) from the list of 1059 genes with de novo chromatin activation using RNA-seq data from 37 MM samples. In this way, we detected 42 pairs and one triplet of coexpressed genes (Supplemental Table S8). From this list, we selected one coregulated chromatin region containing two unrelated genes, *PRDM5* and *NDNF.* PRDM5 is a transcription factor member of the PRDM family that, in contrast to PRDM1, is not expressed in normal plasma cells. Considering that PRDM1 is one of the key regulators of normal plasma cell differentiation, we hypothesized that the aberrant regulation of one of the members of the PRDM family could have a significant impact on MM pathogenesis. Moreover, PRDM5 has been described as regulating gene transcription by recruitment of histone modifier enzymes or other transcription factors (Duan et al. 2007; Shu et al. 2011; Galli et al. 2012; Shu 2015). Therefore, its overexpression could lead to deregulation of the MM transcriptome, leading to the aberrant activation of different signaling pathways. In the case of NDNF, this gene is involved in neuron biology that has not been yet related to any neoplastic phenotype (Kuang et al. 2010; Ohashi et al. 2014). Therefore, the main reason for choosing the *PRDM5-NDNF* pair was because of the potential role of PRDM5 as an oncogene in MM and the fact that *NDNF* expression was highly correlated with that of *PRDM5*. These two coexpressed genes reside in a 500-kb region showing clear de novo activation in MM (Fig. 4A,B), and such coexpression was validated in an additional sample cohort of 10 MM patients and 10 MM cell lines by RT-qPCR (Supplemental Fig. S16A). In addition to their coexpression, both genes were clearly up-regulated in MM, with negligible expression levels across B cell differentiation, including t-PCs and bm-PCs (Fig. 4B). Considering the high correlation between both transcripts, we sought to determine whether *PRDM5* expression could be regulating *NDNF* transcription or vice versa. Targeted gene silencing by shRNA revealed that neither did PRDM5 down-regulation affect NDNF expression nor did NDNF down-regulation alter PRDM5 expression. This and other experiments (Supplemental Fig. S17) did not provide evidence of a direct coregulation between these two genes, suggesting that alternative mechanisms account for their correlated expression. We then studied whether *PRDM5* and *NDNF* co-activation is related to a specific three-dimensional genome organization of the region. We performed a 4C-seq experiment in three MM cell lines expressing both transcripts (i.e., KMS-11, MM.1S, and U266) and a mantle cell lymphoma cell line (JVM-2) as a negative control. Using different viewpoints, we could identify a MM-specific area with increased three-dimensional interactions which contains both *PRDM5* and *NDNF*, suggesting that the topological remodeling of this region in MM may be related to the coordinated activation of both genes (Fig. 4C; Supplemental Fig. S18). Finally, in order to identify whether these physically related but functionally independent genes are involved in MM pathogenesis, we performed doxycycline-inducible shRNA mediated knockdown in different MM cell lines. Gene knockdown at the transcriptional and protein level was validated by RT-qPCR (Fig. 4D; Supplemental Fig. S16B) and western blot (Supplemental Fig. S16E) to ensure the correct silencing of the target gene. *PRDM5* knockdown induced a significant decrease in cell proliferation rate and increased cell death (Fig. 4E,F; Supplemental Fig. S16C,D). However, we did not detect any significant effect in cell viability after *NDNF* silencing (Fig. 4E,F; Supplemental Fig. S16C,D). Taken together, these results suggest that this de novo activation of the coregulated chromatin region comprising *PRDM5* and *NDNF* drives the coordinated overexpression of both genes, but only *PRDM5* seems to be related to MM pathogenesis. This evidence infers that *PRDM5* acts as an oncogene in MM, which contrasts with its previously reported tumor suppressor role in solid tumors (Duan et al. 2007; Shu et al. 2011; Shu 2015). In order to decipher the specific function of *PRDM5* in MM pathogenesis, we performed RNA-seq analysis comparing the transcriptional landscape of a *PRDM5* silenced versus a mock MM cell line. We found 1216 differentially expressed genes (Fig. 4G), suggesting that this transcription factor regulates a complex transcriptional regulatory network, implicated in multiple cellular processes and signaling pathways (such as TNF, IL2-STAT5, KRAS, MTOR, MYC, or TP53). Out of these, we detected that genes down-regulated upon *PRDM5* silencing were associated with the cell cycle as well as various types of cellular stress responses such as DNA damage stress induced by UV light and unfolded protein response stress, suggesting that *PRDM5* may be involved in protecting MM cells against the cellular stress created by proliferation and high protein synthesis (Fig. 4H), although further functional validation is required.

## Discussion

Overall, we provide a multi-omics view of the MM epigenome in the context of normal B cell differentiation which extends recent efforts to analyze polycomb-mediated gene repression and regulatory networks driven by superenhancer elements in MM pathogenesis (Agarwal et al. 2016; Jin et al. 2018). Our series contains cases from different genetic groups, reflecting the heterogeneous nature of the disease. However, the sample size is not informative to characterize such biological variability. Considering such limitation, we decided to focus on identifying common epigenetic events involved in MM pathogenesis by integrating two data sets: first, a multilayered epigenomic characterization of MM patients that provided a global view of the chromatin function and deregulation; and second, an extended series focused on the profiling of the chromatin regulatory landscape and the identification of regulatory elements. By the comprehensive integration of both data series, we could identify common MM-specific signatures for multiple epigenetic marks, revealing the existence of a core epigenomic landscape underlying MM pathogenesis. This was mostly associated with the widespread activation of regulatory elements silenced not only in PCs, the normal counterpart of MM, but across the entire B cell maturation program. Such activation seems to be mediated by the action of specific TF families, such as IRF, FOX, or MEF2, and associated with a de novo loss of DNA methylation in their target regions. These families are part of a TF network deregulated in MM (Jin et al. 2018). In addition, members of these three TF families have been previously reported to be functionally important for MM pathogenesis (Carvajal-Vergara et al. 2005; Shaffer et al. 2008; Campbell et al. 2010; Bai et al. 2017; Agnarelli et al. 2018). Thus, inhibition of these TFs may represent a rational therapeutic approach to revert aberrant chromatin activation in MM. This hypothesis is supported by previous publications indicating that IRF4 or FOXM1 inhibition induces cell death and influences the proliferation in MM cell lines, although efficient strategies to block these TFs in MM patients are still missing (Shaffer et al. 2008; Morelli et al. 2015; Gu et al. 2016).

The altered chromatin landscape in MM affects genes involved in a variety of signaling pathways and cellular responses previously reported to play a central role into myelomagenesis (Bommert et al. 2006; Hu and Hu 2018). In particular, MM cells de novo activate regulatory elements of genes involved in preventing cell death associated with oxidative stress. One of the major systems maintaining cellular redox homeostasis is the thioredoxin system (Arnér and Holmgren 2006). We show that down-regulation of *TXN* expression either by disrupting the gene itself or by deleting its distant regulatory element impairs MM cell growth, representing a potential therapeutic target. In addition to the emergence of enhancer elements, we also identified the activation of coregulated chromatin regions, which coordinate the deregulation of more than one adjacent target gene. This observation resembles the phenomenon of long-range epigenetic activation in cancer, which spans more than one up-regulated gene (Bert et al. 2013), which in turn may be related to changes in topologically associating domains through insulator dysfunction (Flavahan et al. 2016). Therefore, it could be plausible that the widespread epigenetic modulation of MM cells could lead to the disruption of the chromatin topology in these cells, activating not only key oncogenes for this disease but also their neighbor transcripts. In this study, we functionally analyzed the impact of two of these coexpressed genes, *PRDM5* and *NDNF*, and only the former has an impact on MM cell growth. In the case of NDNF, we did not find evidence for an oncogenic role in MM, suggesting that its up-regulation may be a consequence of long-range epigenetic changes initiated by oncogenic PRDM5 activation. However, as we only evaluated the effect on proliferation and apoptosis, we cannot rule out that NDNF may be involved in MM through other functional mechanisms previously reported for this gene in different non-neoplastic contexts, such as cell adhesion, migration, or regulation of angiogenesis (Kuang et al. 2010; Ohashi et al. 2014). Furthermore, through the transcriptional analysis of *PRDM5* knocked-down MM cells, we discovered that this transcription factor seems to regulate a complex transcriptional regulatory network, implicated in cell cycle regulation and different signaling pathways, but also responses to DNA damage and protein synthesis stresses. Supporting our results, a previous report already identified that *PRDM5* is up-regulated upon UV-light exposure, and its promoter contains binding sites of stress-related TFs (Shu et al. 2011); however, further functional validation of the possible role of PRDM5 in stress response is required in MM patient samples. Altogether, our findings support the hypothesis that protection against various cellular stresses is a key element for the survival of MM cells and that neoplastic plasma cells may in part achieve this stress-resistant phenotype through aberrant de novo chromatin activation. From the clinical perspective, a limitation of our report is the sample size, and additional studies in larger series of homogeneously treated patients shall be undertaken to determine whether chromatin activation, either globally or in specific regions, is related to the clinical behavior of the patients. In conclusion, extensive chromatin activation seems to be a unifying principle underlying multiple pathogenic mechanisms in MM, which suggests that epigenetic drugs such as, for example, BET inhibitors, may be appropriate as a backbone treatment for patients affected with this aggressive disease.

## Methods

### Healthy donors, MM patient samples, and cell lines

Purified plasma cells from bone marrow aspirations were obtained from newly diagnosed patients of MM (*n* = 22), with over 90% purity in all cases. The data from normal B cells (i.e., pb-NBC, t-NBC, GCBC, MBC, t-PC) wwere generated previously, using samples collected and isolated as previously described (Kulis et al. 2015; Beekman et al. 2018). All patients and donors gave informed consent for their participation in this study, which was approved by the clinical research ethics committee of Clínica Universidad de Navarra. KMS-11, RPMI8226, MM.1S, and U266 MM cell lines, as well as the JVM-2 cell line were maintained in RPMI-1640 medium (Lonza), following standard cell culture protocols. Further details on the biological materials used in our study are described in the Supplemental Materials.

### Generation of epigenomic data and computational analyses

ChIP-seq for six different histone marks (i.e., H3K27ac, H3K4me1, H3K4me3, H3K36me3, H3K27me3, and H3K9me3) and ATAC-seq were generated as described (http://www.blueprint-epigenome.eu/index.cfm?p=7BF8A4B6-F4FE-861A-2AD57A08D63D0B58), following the high-quality standards of the Blueprint Consortium (EU contribution to the International Human Epigenome Consortium). Whole-genome bisulfite sequencing was carried out following previously established workflows (Kulis et al. 2015). Gene expression of the reference epigenome and the validation series was performed by strand-specific RNA-seq, while in the case of shPRDM5 cell lines, a MARS-seq protocol was adapted for bulk RNA-seq (Jaitin et al. 2014). Details on the experimental procedures of data generation and processing, detection of differential epigenetic regions and de novo active H3K27ac regions, detection of differentially methylated CpGs, differential expression analysis, Gene Ontology, and transcription factor analysis are described in the Supplemental Materials.

### Characterization and functional studies of the *TXN* locus

To map significant 3D interactions of the *TXN* promoter with the identified active enhancer elements in MM, previously published Hi-C data were mined (GSE63525) (Rao et al. 2014). Further validation of the functionality of the *TXN* gene itself and the identified candidate regulatory elements in MM cells was performed using CRISPR-Cas9 assays. The generation of CRISPR-Cas9 constructs and the production of lentivirus were performed according to standard protocols. CRISPR-Cas9 editing efficiency was determined by different techniques, including PCR assays, Sanger sequencing, and next-generation sequencing (NGS). mRNA and protein levels of TXN after CRISPR-Cas9 editing were assessed by RT-qPCR and western blot analyses, respectively. gRNA and primer sequences used are in Supplemental Table S9. In the pool of edited cells, changes in proliferation (using a fluorescent endogenous reporter), apoptosis (FITC Annexin V Apoptosis Detection kit I, BD Biosciences), or ROS levels (CellROX Deep Red Reagent, Thermo Fisher Scientific) were monitored by flow cytometry assays. Detailed protocols for each technique are described in Supplemental Materials.

### Functional characterization of the *PRDM5* and *NDNF* loci

4C-seq templates for KMS-11, U266, MM1.S, and JVM-2 cell lines were prepared as previously described (Simonis et al. 2007; van de Werken et al. 2012). We performed this experiment for the *NDNF* promoter (Chr 4: 121,070,660–121,071,025), *PRDM5* region (Chr 4: 120,816,668–120,817,023), and an additional negative control viewpoint (Chr 4: 121,234,271–121,234,614), using DpnII and BfaI as first and second restriction enzymes and the primers listed in Supplemental Table S9. Data analysis was performed with the 4Cseqpipe pipeline with default settings and removing reads corresponding to self-ligated or nondigested fragments.

shRNA-mediated knockdown of both *PRDM5* and *NDNF* genes was performed using a Tet-On inducible lentiviral system. Design and generation of shRNA constructs and lentiviral production were performed according to standard protocols. shRNA expression was induced by adding 1 μg/mL doxycycline (Sigma-Aldrich) to the culturing media. Target gene knockdown was validated by RT-qPCR and western blot assays. Cell viability in the cell pool was measured by MTS assay, and apoptosis levels were determined by flow cytometry (FITC Annexin V Apoptosis Detection kit I, BD Biosciences).

To characterize a possible reciprocal activation between *PRDM5* and *NDNF*, *PRDM5* expression was determined by RT-qPCR upon *NDNF* silencing and vice versa. Additionally, the PRDM5 binding motif was calculated using publicly available data (Galli et al. 2012, 2013) and mapped to the *NDNF* promoter region, identifying one putative binding site. A luciferase reporter assay was then used to assess the potential role of the PRDM5 transcription factor in *NDNF* overexpression. Detailed experimental procedures are described in Supplemental Materials.

## Data access

The raw data on MM and normal B cells included in this study have been submitted to the European Genome-Phenome Archive (EGA; https://ega-archive.org), which is hosted at the European Bioinformatics Institute (EBI) under accession numbers EGAS00001000326 (ChIP-seq), EGAS00001001596 (ATAC-seq), and EGAS00001000418 (WGBS) and released as part of the BLUEPRINT epigenome project. Additional series of MM RNA-seq data generated in this study have been submitted to the NCBI Gene Expression Omnibus (GEO; https://www.ncbi.nlm.nih.gov/geo/) under accession number GSE151063. Furthermore, we have created a website (http://resources.idibaps.org/paper/chromatin-activation-as-a-unifying-principle-underlying-pathogenic-mechanisms-in-multiple-myeloma) that includes the large processed data matrices and a link to a UCSC Genome Browser session displaying the generated data.

## Competing interest statement

The authors declare no competing interests.

## Supplementary Material

Supplemental Material
