# Supplementary material for: Chromatin activation as a unifying principle underlying pathogenic mechanisms in multiple myeloma
Source: Genome Res. 2020 Sep;30(9):1217–27. doi: 10.1101/gr.265520.120 (PMC7545147; doi:10.1101/gr.265520.120)
Supplement: Supplemental Material [file supp_30_9_1217__index.html]

Chromatin activation as a unifying principle underlying pathogenic mechanisms in multiple myeloma — Supplemental Material 

# Chromatin activation as a unifying principle underlying pathogenic mechanisms in multiple myeloma

## Supplemental Material

- Supplemental\_Table\_S3.xlsx
- Supplemental\_Table\_S4.xlsx
- Supplemental\_Table\_S5.xlsx
- Supplemental\_Table\_S6.xlsx
- Supplemental\_Table\_S7.xlsx
- Supplemental\_Table\_S8.xlsx
- Supplemental\_Table\_S9.xlsx
- Supplemental\_Table\_S1.xlsx
- Supplemental\_Table\_S2.xlsx
- Supplementary\_materials\_Ordonez-Kulis\_final.pdf
