# Supplementary material for: Chromatin activation as a unifying principle underlying pathogenic mechanisms in multiple myeloma
Source: Genome Res. 2020 Sep;30(9):1217–27. doi: 10.1101/gr.265520.120 (PMC7545147; doi:10.1101/gr.265520.120)
Supplement: Supplemental Material [file supp_gr.265520.120_Supplementary_materials_Ordonez-Kulis_final.pdf]

SUPPLEMENTAL MATERIALS

Chromatin activation as a unifying principle underlying pathogenic mechanisms in multiple myeloma

Ordoñez&Kulis et al.

METHODS.. 3
Collection and preparation of patient and normal samples.. 3
ChIP-seq, ATAC-seq, RNA-seq and WGBS data generation.. 4
Read mapping and initial data processing. 5
Detection of differential epigenetic regions and *de novo* active regions. 7
Detection of differentially methylated CpGs. 8
Differential expression analysis 8
Gene Ontology and canonical pathways analysis of data sets 9
Transcription factor analysis 9
Cell culture 9
3D analysis of *TXN* locus.. 10
4C-seq data generation and analysis 10
Inducible shRNA knockdown system and lentiviral production. 11
TXN editing by CRISPR-Cas9 system.. 11
CRISPR-Cas9 editing validation by NGS... 12
Expression analysis by RT-qPCR 13
Luciferase assay.. 13
Western blot 14
Apoptosis assay.. 14
Cell viability assays by MTS 14
Cell proliferation assays by flow cytometry 15
Measurement of reactive oxygen species (ROS) by flow cytometry. 15
Supplemental References 15

SUPPLEMENTAL FIGURES 18
Supplemental Figure S1 18
Supplemental Figure S2 19
Supplemental Figure S3 20

Supplemental Figure S4 21
Supplemental Figure S5 22
Supplemental Figure S6 .... .23
Supplemental Figure S7 24
Supplemental Figure S8 25
Supplemental Figure S9 26
Supplemental Figure S10 27
Supplemental Figure S11 28
Supplemental Figure S12 29
Supplemental Figure S13 30
Supplemental Figure S14 31
Supplemental Figure S15 32
Supplemental Figure S16 33
Supplemental Figure S17.......... 35
Supplemental Figure S18 37

SUPPLEMENTAL TABLES 38

METHODS

Collection and preparation of patient and normal samples. Tumor samples used for epigenomic analyses were obtained from bone marrow aspirations of newly diagnosed patients of MM before administration of any treatment. All MM samples were purified by CD138+ positive magnetic separation using AutoMACS system (AutoMACS Pro Separator, Miltenyi Biotech), obtaining over 90% purity in all cases, as assessed by flow cytometry. The data from normal B cells (i.e. pb-NBC, t-NBC, GCBC, MBC, t-PC) was generated by our lab previously, using samples collected and isolated as previously described (Kulis et al. 2015; Beekman et al. 2018). Purified plasma cells (bm-PC) were selected from bone marrow aspirations from healthy donors ranging from 20 to 30 years. After Ficoll-Isopaque density gradient centrifugation, we performed a selective depletion of CD3+, CD14+ and CD15+ cells by immunomagnetic selection (Miltenyi Biotec), followed by FACS of CD45+ / CD138+ / CD38+ cells using a FACSAriaII (BD Biosciences) device (**Supplemental Table S1**).

For downstream transcriptional analyses, an additional series of MM cases and normal B cell controls was studied. Tumor samples were obtained and purified following the same strategy as described above, while naive B cells (NBC), germinal center B cells (GCBC), memory (MBC) and tonsillar plasma cells (t-PC) were isolated from human tonsils of healthy donors while bone marrow plasma cells (bm-PC) from bone marrow of healthy donors by multiparameter fluorescence-activated cell sorting (FACS) using the expression level of nine different surface antigens as previously described (Pascual et al. 2016). The following monoclonal antibody (MoAb) combination was used for the cell isolation from tonsils: CD45-OC515 (Clone HI30, Immunostep, Salamanca, Spain); CD20-Pacific Blue (Clone 2H7, Bioloegend, San Diego, California, UnitedStates); CD44-APCH7 (Clone G44-26, Beckton Dickinson, Dur-ham, North Carolina, United States). CD10 PE-Cy7 (Clone HI10a BecktonDickinson, Durham, North Carolina, United States); CD38-FITC (Clone LD38, Cytognos, Salamanca, Spain); CXCR4-PE (Clone 12G5, Beckton Dickinson, Durham, North Carolina, UnitedStates); CD27-APC (Clone L128, Beckton Dickinson, Durham, North Carolina, UnitedStates) and CD3-PerCP-Cy5.5 (Clone SK7, Beckton Dickinson, Durham, North Carolina, United States). bm-PC were sorted using FACS AriaIIfrom human bone marrow of healthy donors using CD38-FITC (Clone LD38, Cytognos, Salamanca, Spain); CD138-BV421 (Clone MI15, Beckton Dickinson, Dur-ham, North Carolina, United States) and CD27-BV510 (Clone 0323, Bioloegend, San Diego, California, UnitedStates) (**Supplemental Table S1**).

All patients and donors gave informed consent for their participation in this study, which was approved by the clinical research ethics committee of Clínica Universidad de Navarra.

ChIP-seq, ATAC-seq, RNA-seq and WGBS data generation. ChIP-seq of the six different histone marks and ATAC-seq data were generated as described (http://www.blueprint-epigenome.eu/index.cfm?p=7BF8A4B6-F4FE-861A-2AD57A08D63D0B58), following the high quality standards of Blueprint Consortium (EU contribution to the International Human Epigenome Consortium). Catalog numbers of antibodies (Diagenode) used are H3K27ac: C15410196/pAb-196-050 (LOT: A1723-0041D), H3K4me1: C15410194/pAb-194-050 (LOT: A1863-001P), H3K4me3: C15410003-50/pAb-003-050 (LOT: A5051-001P), H3K36me3: C15410192/(pAb-192-050 (LOT: A1847-001P), H3K9me3: C15410193/pAb-193-050 (LOT: A1671-001P), H3K27me3: C15410195/pAb-195-050 (LOT: A1811-001P). In order to minimize any technical problems (i.e. Ab specificity), all these antibodies were broadly tested and validated from different perspectives before being applied for ChIP-Seq experiments. All the data concerning the validated Blueprint antibodies as well as established SOPs are publicly available on Blueprint-Epigenome website (http://www.blueprint-epigenome.eu/index.cfm?p=D6F8811F-DACF-7979-CEAC0B9034C28037).

Strand specific RNA-seq data of the reference epigenomes and the validation series was generated as previously described (Ecker et al. 2017). Briefly, RNA was extracted using TRIZOL (Life Technologies) and libraries were prepared using a TruSeq Stranded Total RNA Kit with Ribo-Zero Gold (Illumina). Adapter-ligated libraries were amplified and sequenced using 100bp single-end reads for the reference epigenome samples and 50bp paired-end reads for the samples in the validation series.

RNA-seq data for the shPRDM5 cell lines series was performed following MARS-seq protocol adapted for bulk RNA-seq (Jaitin et al. 2014) with minor modifications. Briefly, 150,000 cells were harvested in 100 ul of Lysis/Binding Buffer (Ambion), vortexed and stored at -80ºC until further processing. Poly-A RNA was reverse-transcribed using poly-dT oligos carrying a 7 bp-index. Pooled samples were subjected to linear amplification by IVT. Resulting RNA was fragmented and dephosphorylated. Ligation of partial Illumina adaptor sequences was followed by a second RT reaction. Full Illumina adaptor sequences were added during final library amplification. RNA-seq libraries quantification was done with Qubit 3.0 Fluorometer (Life Technologies) and size profiles examination with Agilent's 4200 TapeStationSystem. Libraries were sequenced in an

Illumina NextSeq 500 at a sequence depth of 10 million reads per sample. Raw data was deposited in GEO, under accession number GSE122638.

WGBS of the reference epigenomes was generated as previously described (Kulis et al. 2015). Briefly, 1–2 µg of DNA was sheared and fragments of 150–300 bp were selected using AMPure XP beads (Agencourt Bioscience). After adaptor ligation (Illumina TruSeq Sample Preparation kit), DNA was treated with sodium bisulfite using the EpiTexy Bisulfite kit (Qiagen). Two rounds of bisulfite conversion were performed to ensure a conversion rate of over 99%. Enrichment for adaptor-ligated DNA was carried out through seven PCR cycles and paired-end DNA sequencing (2 × 100 bp) was then performed using the Illumina HiSeq 2000 platform.

Read mapping and initial data processing. Fastq files of ChIP-seq data were aligned to genome build GRCh38 (using bwa 0.7.7, picard 2.8.1 and samtools 1.3.1) and wiggle plots were generated (using PhantomPeakQualTools 1.1.0) as described (http://dcc.blueprint-epigenome.eu/#/md/methods).

Peaks of the histone mark data were called as described (http://dcc.blueprint-epigenome.eu/#/md/methods) using MACS2 (version 2.1.1.20160309), with input control. ATAC-seq fastq files were aligned to genome build GRCh38 using bwa 0.7.759 (parameters: -q 5, -P, -a 480) and SAMTOOLS v1.3.160 (default settings). BAM files were sorted and duplicates were marked using PICARD tools v2.8.1 (http://broadinstitute.github.io/picard, default settings). Finally, low quality and duplicates reads were removed using SAMTOOLS v1.3.160 (parameters: -b, -F 4, -q 5, -b, -F 1024). ATAC-seq peaks were determined using MACS2 (v2.1.1.20160309, parameters: -g hs -q 0.05 --keep-dup all -f BAM –nomodel –shift -96 –extsize 200) without input control. For downstream analysis peaks with p-values <1e-3 (H3K36me3, H3K9me3 and H3K27me3) or <1e-7 (H3K4me3, H3K4me1, H3K27ac, ATAC-seq) were included. For each mark a set of consensus peaks, only including regions on chromosome 1-22, present in the normal B cells (n=15 for histone marks and n=18 for ATAC-seq) and in the MM samples (n=4 for H3K4me3, H3K36me3, H3K9me3, H3K27me3; n=14 for the H3K27ac series, n=10 for the H3K4me1 series and n=17 for the ATAC-seq series) was generated by merging the locations of the separate peaks per individual sample. To generate the consensus peak file for the reference epigenomes, only peaks with input were used except for ATAC-seq for which peaks without input were used. For ChIP-seq, the numbers of reads per sample per consensus peak were calculated using the genomecov function of bedtools. For the ATAC-seq, the number of insertions of the Tn5 transposase per sample per consensus peak were calculated by first determining the estimated

insertion sites (shifting the start of the first mate 4bp downstream), followed by the genomecov function of bedtools. Using DESeq2 (Anders and Huber 2010), variance stabilizing transformation (vst) values were calculated for all consensus peaks. The number of consensus peaks per chromatin mark were 72,848 (H3K4me3), 60,494 (H3K4me1), 125,026 (H3K27ac), 33,907 (H3K36me3), 61,142 (H3K9me3), 34,499 (H3K27me3), and 121,860 (ATAC-seq). Principal component analyses (PCA) were generated with the prcomp function in R using the (corrected) vst values of all peaks that were present in >1 sample, thus eliminating possible individual-specific peaks.

ChIP-seq of H3K27ac in bm-PC was processed as described above and peaks were called as described (http://dcc.blueprint-epigenome.eu/#/md/methods) using MACS2 (version 2.1.1.20160309).

Three different variants of RNA-seq were performed, which were processed as follows: 1) RNA-seq data of the reference epigenome samples (3 MM and 15 normal B cells) was aligned to genome build GRCh38, signal files were produced and gene quantifications (gencode 22; 19,736 protein coding genes) were calculated as described (http://dcc.blueprint-epigenome.eu/#/md/methods) using the GRAPE2 pipeline with the STAR-RSEM profile (adapted from the ENCODE Long RNA-seq pipeline). The expected counts and FPKM estimates were used for downstream analysis. The PCA of the RNA-seq data was generated with the prcomp function in R using log10 transformed FPKM (+0.01 pseudocount) data. 2) RNA-seq of additional validation series (37 MM and 22 normal B cells; Agirre et al. 2019) was aligned in a two-step procedure, using STAR v2.4 to remove any potential ribosomal RNA leftover from the ribodepletion step. A first pass alignment was done against human ribosomal sequences allowing multi-mapping. Unmapped reads from the first step were then aligned to hg19 human reference using GENCODE v19 junction points. Cufflinks v2.2.1 was run on the resulting alignment files to create *de novo* transcriptome assembly specific to each sample using strand-specific settings and GENCODE v19 as a database. Cufflinks outputs for all of the samples were then merged with themselves and GENCODE database using cuffmerge. R and GNU parallel were used to facilitate the analysis. 3) Low input 3' end RNA-seq data of shPRDM5 knockdown cells was aligned to the GRCh38/hg38 human genome with STAR version 2.5.2B with default parameters. To assign reads to genes we applied feature Counts.

Mapping and determination of DNA methylation estimates was performed using GEMBS, as described by authors (Merkel et al. 2018). We retained 9,214,561 CpGs (chr1-22) showing at

least 10 reads in all 20 samples. All downstream analyses were performed with this DNA methylation matrix.

Detection of differential epigenetic regions and *de novo* active regions. For individual histone marks and ATAC-seq data, only consensus regions present in at least 1 and in a maximum of all but 1 sample were used, thus excluding individual specific and constitutive regions. Of the included consensus peaks, those differentials among MM as compared to normal B cell subpopulations were defined using the likelihood ratio test (FDR < 0.01) of the DEseq2 package. Next, we separated the regions with either stable or dynamic patterns throughout normal B cell differentiation. For that purpose, we performed differential analysis within normal B cell subpopulations by DEseq2 package, classifying those with FDR<0.01 as regions harbouring epigenetic mark modulation throughout normal B cell maturation, and the regions with FDR>0.01 as well as those that have no peaks at all in normal B cells as regions with stable chromatin during B cell maturation. The latter list was used to identify regions with either gain or loss of a particular epigenetic mark in MM (**Supplemental Fig. S1**).

To stringently identify regions *de novo* active in MM, several filters were applied to the previously detected set of regions with differential gain of H3K27ac (n=12,195). This strategy is depicted in **Supplemental Figure S7**. First, the H3K27ac profile of bm-PC from one healthy donor was generated and the peaks shared between MM and bm-PC were filtered out. Next, the percentages of H3K27ac peak occupancy within each differential region were calculated and those regions having less than 20% of H3K27ac in normal B cells while showing a consistently present H3K27ac gain in MM (FDR<0.05) were selected. Finally, in order to identify the regions with an impact on gene expression, we performed the following steps: 1) *de novo* H3K27ac regions were annotated to all the genes lying within the same topological associated domain (TAD) in the GM12878 lymphoblastoid cell line, a well-established cellular model for malignancies of the B cell lineage. TADs were defined by the TADbit workflow (as described in Beekman et al. 2018 and Serra et al. 2017) using publicly available Hi-C data (GSE63525; Rao et al. 2014); 2) gene expression changes between MM and normal B cells were identified by comparing RNA-seq data from 37 MM cases and 22 normal mature B cell subtypes samples, including 3 also healthy bm-PCs (data from additional validation RNA-seq series); and 3) assuming that the target genes of *de novo* active regulatory regions within a TAD would be overexpressed, only the genes overexpressed in MM as compared to all normal B cells (Limma, FDR<0.05) and with clearly higher expression in MM versus bm-PC (|FC| >1.5) were selected.

This strategy led to the identification of 1,059 target genes corresponding to 1,556 regions with *de novo* gain of H3K27ac in MM. The identify regions and target genes *de novo* repressed in MM, the same type of analysis was performed with all the analogous steps but for the contrary situation. In this case, only 4 repressed regions were identified, corresponding to 6 genes downregulated in MM.

Super-enhancers within detected 1,556 *de novo* active chromatin regions were defined either by stitching peaks using ROSE (Whyte et al. 2013; Loven et al. 2013) with default parameters (except TSS exclusion) or by selecting H3K27ac regions exceeding 10kb in size.

Detection of differentially methylated CpGs. Detection of differentially methylated CpGs (DMCs) in MM as compared to normal B cells was performed using limma with DNA methylation difference between conditions of 0.25 and FDR <0.05. Furthermore, detection of DNA methylation valleys (DMVs) was performed as described by Wei Xie et al. (Xie et al. 2013). Briefly, we employed a window-based approach to determine DMVs. To identify each DMVs in each sample, the genome was first divided in 1kb bins and the DNA methylation level was averaged within each bin. Then a sliding 5kb window (with 1kb step) was used to identify regions that have a median methylation level less than 0.15 in a 5kb window in MM samples. Continuous regions resulting from this analysis were then merged to form DMVs in each sample.

Differential expression analysis. In this study, three different types of RNA-seq were analyzed, depending on the purpose of the study, i.e. RNA-seq of the reference epigenome samples, RNA-seq of additional validation series and low input RNA-seq for characterization of transcriptional changes in samples from PRDM5 knockdown studies. For the first two RNA-seq experiments, Limma analysis was performed in order to obtain differentially expressed protein-coding genes (FDR < 0.05, |FC|>1.5) between MM samples and all B cell subpopulations. The threshold to consider a gene as unexpressed was set to 0.1 FPKM. For the low input 3' end RNA-seq dataset of shPRDM5 knockdown cells, only genes that were expressed (sum of FPKM values in all samples greater than 10) were included. Differential expressed genes were defined using the likelihood ratio test of DEseq2 package (|FC|> 1.5 and p < 0.01).

Gene Ontology and canonical pathways analysis of data sets. GO enrichment was performed using GOrilla online tool (Eden et al. 2009), and further summarized together by REVIGO software (Supek et al. 2011) in order to represent them as semantic-similarity scatter plot of the most significant GO terms. Hallmark gen set analysis was performed, using MSigDB Collections platform (Liberzon et al. 2015; Subramanian et al. 2005). Additionally, Gene Set Enrichment Analysis (GSEA) was performed using standard settings, with ranking according to p-values. We applied two different GSEA collections: biological processes sets (c5.bp) and pathways sets (c2.cp), downloaded from MSigDB Collections platform.

Transcription factor analysis. Enrichment for TF binding sites was analyzed in regions gaining chromatin accessibility within the 1,556 *de novo* active regions in MM. Gain of ATAC-seq peaks was determined as regions without any ATAC-peak in the normal B cells (n=15) while showing a significant gain of chromatin accessibility in the MM cohort (n=17; FDR<0.01). Using this strategy, we ended up with 806 sites. Enrichment analysis of known TF binding motifs was performed using the AME tool from MEME suite (McLeay and Bailey 2010), from the non-redundant homo sapiens 2018 Jaspar database (537 TF motifs), applying a one-tailed Wilcoxon rank-sum test with the maximum score of the sequence, a 0.05 FDR cutoff and a background formed by reference GRCh38 sequences extracted from the consensus ATAC-seq peaks enriched in at least two samples.

For the study of DNA methylation levels associated with TF binding sites of three main TF families in MM, we applied the FIMO application from MEME suite. In the case of IRF, we identified 49 CpGs located within the motifs, while for FOX and MEF2 we found 84 and 90 CpGs, respectively, in their close proximity i.e. 50 bp upstream and downstream of their detected binding sites. We studied CpGs methylation status using WGBS data in 12 normal B cells and 5 MM samples, defining as differential the ones with absolute methylation difference above 0.25.

Cell culture. KMS-11, RPMI8226, MM.1S and U266 MM cell lines, as well as JVM-2 cell line were maintained in RPMI-1640 medium (Lonza) supplemented with 20% FBS (Gibco), 1% Penicillin / Streptomycin (Lonza) and 2% HEPES (Life Technologies). All cells were maintained at 37 ^{o}C and 5% CO_2. HEK293T cells were maintained in DMEM (Lonza) with high glucose and pyruvate, supplemented with 10% FBS (Gibco), 1% Penicillin / Streptomicyn (Lonza) and 2% HEPES (Life

Technologies) and grown in a humidified atmosphere at 37 °C and 5% CO_2. Only cells at low passage (below passage 16) were used for lentiviral production.

3D analysis of *TXN* locus. In order to identify 3D interactions of the *de novo* active enhancer regions close to *TXN*, we used published Hi-C data of the GM12878 lymphoblastoid cell line (GSE63525; Rao et al. 2014), in which the chromatin architecture of the *TXN* locus is similar to MM cases. We used the following approach: 26 Hi-C samples of GM12878 were processed and merged by the 4Dgenome unit, obtaining over 3 billion of valid reads using the TADbit pipeline. Briefly, high quality reads were aligned to the reference genome (version GRCh38), applying a fragment-based strategy. Non-informative reads were filtered out using the default parameters of TADbit and the remaining reads were merged into a single Hi-C dataset. Initially, for each of the two adjacent regulatory regions of interest, we determined their interaction frequencies throughout whole chromosome 9 as a function of distance. We observed that for both regions, the vast majority of the interactions occur at a distance shorter than 100kb. *TXN* is located around 80kb from our enhancer regions, therefore we decided to investigate the top interactions within the expanded region of 160kb upstream and downstream of our region of interest (240 kb in total). The analysis was performed at 2kb resolution and once we corrected the interactions by the global decay, we selected the contacts within the top 5% fold-change.

4C-seq data generation and analysis. 4C templates for JVM-2, KMS-11, MM.1S and U266 cell lines were prepared as previously described (Simonis et al. 2007; van de Werken et al. 2012), using 10^7 cells per 4C library. We performed this experiment for three different viewpoints: 1) the *NDNF* promoter (chr4: 121,070,660-121,071,118) using DpnII and BfaI as first and second restriction enzymes and the following respective primers: *5'-TTGCTTCTCATCTGTCGATC-3' and 5'-CAGAAAGGTGAACCGAGAG-3';* 2) the *PRDM5* region (chr4: 120,816,668-120,817,023) using DpnII and BfaI as first and second restriction enzymes and the following respective primers: 5'-ATAAAGTCATGTTCGTGATC-3' and 5'-CATGCATACTTTCATGCTTC-3' and 3) negative control region (chr4: 121,234,271-121,234,614, proximity of *TNIP3* gene) using DpnII and BfaI as first and second restriction enzymes and the following respective primers: 5'-GGGGTCTCCAAACTGAGATC-3' and 5'-TACCTCCTAAATGCTTTCAAC-3'. Data analysis was performed with the 4Cseqpipe pipeline using default settings and removing reads corresponding to self-ligated or non-digested fragments.

Inducible shRNA knockdown system and lentiviral production. Short hairpin RNAs (shRNA) against each of the genes of interest (i.e. *PRDM5* or *NDNF*) were designed using public available algorithms (http://www.broad.mit.edu/science/projects/rnai-consortium/trc-shrna-design-process and https://www.med.nagoya-u.ac.jp/neurogenetics/i_Score/i_score.html). shRNAs were cloned into a TET inducible version of pLKO.1 vector (Tet-pLKO-puro, Addgene #21915) after EcoRI/AgeI digestion. A shRNA targeting GFP gene (shGFP) was used as experimental control. See **Supplemental Table S9** for target sequences and sequencing primers.

Lentiviruses were generated by co-transfecting HEK293T cells with shRNA-encoding plasmid, psPAX2 (Addgene, #12260) and pMD2G (Addgene, #12259) plasmids in a 3:2:1 ratio, using Lipofectamine 2000 (Invitrogen). Growth media was exchanged after 12 hours. Lentivirus-containing supernatant was harvested 48 hours later, filtered (0.2 µm) and applied directly to cells for infection with 2 µg/ml polybrene (Sigma). Target cell lines were selected in 1.5 µg/ml puromycin (Sigma) for 72 hours.

Stable cell lines harboring the silenced shRNA-expressing cassette were grown in Tet-Free FBS (Fetal Bovine Serum (FBS) South America, Tetracycline Free, Biowest) conditions to prevent Tet-promoter basal expression. shRNA expression was induced by adding 1 µg/ml doxycycline (Sigma) to the culturing media and replaced every 48 hours. Target gene knockdown was validated by RT-qPCR to ensure the correct silencing of the target gene.

***TXN* editing by CRISPR-Cas9 system.** To perform CRISPR-Cas9 editing of *TXN* gene, we first generated two stable Cas9 cell lines: KMS-11 and MM.1S, infecting them with lentiviruses carrying the Cas9-2A-Blasticidin expressing cassette (Lenti-Cas9-2A-Blast plasmid, Addgene #73310) following standard protocol, described before, and selecting them with 10 µg/ml blasticidin (Invitrogen) for 7 days. For *TXN* knockout, we designed two different gRNAs (gTXN#1 and gTXN#2), both targeting exon 2 of the gene, and we cloned them into two separetedly in CRISPseq-BFP-backbone (gift from Ido Amit laboratory, Addgene #85707). See **Supplemental Table S9** for target sequences and sequencing primers. In case of genomic deletion of *TXN* 11kb enhancer region, paired gRNAs flanking the region of interest were designed and cloned separately: one into CRISPseq-BFP-backbone harboring a BFP reporter gene and the other into pLKO5.sgRNA.EFS.GFP (Addgene #57822) harboring a GFP reporter. See **Supplemental Table S9** for target sequences. Corresponding lentiviruses were produced for all the constructs (as

described above), and constitutively Cas9 expressing cell lines were infected. BFP^+ cells in the case of single gRNAs and double BFP^+ / GFP^+ cells in the case of double gRNAs were determined by flow cytometry (FACS Canto II, BD Biosciences) 72 hours after infection. Percentage of BFP^+ cells and BFP^+ / GFP^+ exceeded 90% in all cases (**Supplemental Fig. S13A**). As a control, cells infected with empty CRISPseq-BFP-backbone and/or pLKO5.sgRNA.EFS.GFP were used (scramble, Scr).

To assess editing efficiency in the TXN knockout experiment, genomic DNA was extracted 72h after infection from the total pull of cells, targeted loci were amplified and sequenced by Sanger reaction (for primer sequences see **Supplemental Table S9**). The spectrum and frequency of targeted mutations generated in the cell pool was determined by the public available software TIDE (Brinkman et al. 2018). In the case of *TXN* enhancer deletion, excision was assessed qualitatively by PCR using primers flanking the genomic target region, such that the edited alleles should produce a shorter PCR product than the uncut alleles in the total pull of cells, 72h after infection (**Supplemental Fig. S13B**). For a more quantitative determination of the editing efficiency in the total pull of cells, qPCR was performed using a primer internal to the deleted region, which will only amplify the wild type allele (**Supplemental Table S9**). For statistical analysis, normality test for each group were performed using Shapiro-Wilk. Student *t*-test was used for parametric group comparisons, whereas Mann-Whitney test was used for non-parametric comparisons. Protein levels of target genes were assessed by western blot analyses.

CRISPR-Cas9 editing validation by NGS. To further characterize genome editing efficiency and variant distribution in CRISPR-Cas9 experiments, *TXN* targeted locus was amplified by PCR to generate barcoded libraries that were further analyzed by next-generation sequencing (NGS) in 3 replicates for each gRNA and each MM cell line. Briefly, 50 ng of genomic DNA was first amplified using specific primers (**Supplemental Table S9**) using KAPA HiFi HotStart ReadyMix PCR Kit (Kapa Biosystems) and purified using Agencourt AMPure XP system (Beckman Coulter). Then, nested PCR was performed using 10 ng of PCR mix and universal primers with specific barcodes for each sample were used to generate Illumina amplicons (**Supplemental Table S9**). PCR products were purified as described above. Final library was made mixing equal amounts of the second PCR products and sequenced on Illumina MiSeq (2 × 250 bp paired-end) at >20,000 × coverage at amplified regions. Data were processed according to standard Illumina sequencing analysis procedures. Processed reads were aligned to the reference (GRCh38/hg38) using Bowtie. Reads that did not map to the reference were discarded. Insertions and/or

deletions were determined by comparison of reads against reference using CrispRVariants R-based toolkit (Lindsay et al. 2016).

Expression analysis by RT-qPCR. RNA was isolated using TRIzol reagent (Life Technologies) following the manufacturer's instructions. RNA was retrotranscribed into cDNA using PrimeScript RTPCR Kit (Clontech, Takara) and RT-qPCR was performed using SYBR Green PCR Master Mix (Applied Biosystems) in QuantStudio Real Time PCR System (Thermo Fisher Scientific), following standard protocols. See **Supplemental Table S9** for primer sequences. For statistical comparisons, normality analyses for each group were performed using Shapiro-Wilk test, followed by Levenne test for homogeneity of variances. For parametric group comparisons one-way ANOVA was used, and Kruskall-Wallis test was selected for non-parametric analyses. For multiple-comparisons post-hoc, Tukey correction was used for samples with homogenous variances, Tamhane's T2 for variance heterogeneity and Fisher's LSD with Bonferroni correction for comparisons among 3 groups.

Luciferase assay. To characterize a possible reciprocal activation between PRDM5 and NDNF genes, PRDM5 consensus binding sequence was calculated from publicly available data (Galli et al. 2012; Galli et al. 2013) and mapped it to *NDNF* promoter region (-1.5 kb from TSS) and gene body. One putative PRDM5 binding site (GGAGAGCCGG) was identified in *NDNF* promoter region. A luciferase reporter assay was then used to assess the potential role of PRDM5 transcription factor in NDNF overexpression. Briefly, one kb fragment from the *NDNF* promoter containing a predicted PRDM5 binding site (-679 to +321 bp from the NDNF TSS) was synthesized by GenScript. This fragment was cloned into a pGL4.17[luc2/Neo] vector (Promega #9PIE672) upstream the luciferase reporter gene after NheI / XhoI digestion and named as NDNF-promoter-wild type (NDNFpr-WT). Its counterpart harboring a deletion of PRDM5 binding site (+59 to +72 bp from the NDNF TSS) was synthesized by mutagenesis from the original NDNFpr-WT and named as NDNF- promoter-mutated (NDNFpr-Mut).

HEK293T cells expressing two different shRNAs targeting PRDM5 (**Supplemental Table S9**) were co-transfected with 10 ng/µl of reporter plasmids containing the NDNFpr-WT, NDNFpr-Mut or empty pGL4.17[luc2/Neo] plasmid and 0.5 ng/µl renilla luciferase vector (pRL-SV40 Renilla Luciferase Control Reporter Vector, Promega). shRNA expression was induced by adding 1 µg/ml doxycycline to the culturing media. Forty-eight hours after transfection PRDM5 knockdown was

validated by RT-qPCR and luciferase/renilla activity was measured by a dual luciferase reporter assay (Promega) in an automatic 96-well plate reader according to manufacturer's instructions.

Western blot. For all the western blot presented in this work, cell protein extracts were obtained by lysis buffer containing 1% Triton X-100, 150 mM NaCl, 50 mM Tris [pH 8], supplemented with 1x protease inhibitor cocktail (Complete Mini, Roche), 10 mM NaF and 1 mM sodium orthovanadate for 30 minutes at 4 °C and quantified using BCA (Pierce BCA Protein Assay Kit, Thermo Fisher Scientific) or Bradford (Protein Assay Dye Reagent Concentrate, BioRad) colorimetric assays. Primary antibodies and specific conditions used were: anti-PRDM5 (sc-376277; Santa Cruz Biotechnologies; 1:2,000); anti-TXN (#2429; Cell Signalling; 1:1,000) and as a loading control anti-β-actin (A5441; Sigma; 1:5,000).

Apoptosis assay. Cells were harvested and stained with FITC Annexin V Apoptosis Detection Kit I (BD Biosciences) following the manufacturer's protocol at indicated time points. The percentage of Annexin V^+ cells was analyzed using FACS Canto II (BD Biosciences) and FlowJo flow cytometry analysis software. For statistical comparisons, normality analyses for each group were performed using Shapiro-Wilk test, followed by Levenne test for homogeneity of variances. For parametric group comparisons one-way ANOVA was used, and Kruskall-Wallis test was selected for non-parametric analyses. For multiple comparisons, Tukey correction was used for samples with homogenous variances, Tamhane's T2 for variance heterogeneity and Fisher's LSD with Bonferroni correction for comparisons among 3 groups.

Cell viability assays by MTS. Cell lines infected with viral shRNA vectors were seeded at 40,000 cells/well in 96 well plates in presence or absence of doxycycline. The number of viable cells in proliferation was determined by MTS assay (CellTiter 96 Aqueous One Solution Cell Proliferation Assay, Promega) following the manufacturer's protocol, at indicated time points. Absorbance was measured at $\lambda = 490$nm. Cell viability in each time point was calculated as the percentage of total absorbance cells in each condition/absorbance of control cells. For statistical comparisons, normality analyses for each group were performed using Shapiro-Wilk test, followed by Levenne test for homogeneity of variances. For parametric group comparisons one-way ANOVA was used, and Kruskall-Wallis test was selected for non-parametric analyses. For

multiple comparisons, Tukey correction was used for samples with homogenous variances and Tamhane's T2 for variance heterogeneity.

Cell proliferation assays by flow cytometry. Cell proliferation rate of MM cell lines edited by CRISPR-Cas9 system was monitored by flow cytometry. 72h after gRNA transduction, 100,000 BFP^+ (for single gRNAs system) or double BFP^+ / GFP^+ cells (for paired gRNAs system) were mixed with 100,000 wild type (WT, BFP^- / GFP^-) cells. The percentage of BFP^+ or BFP^+ / GFP^+ cells was quantified by flow cytometry (FACS Canto II, BD Biosciences) and monitored every 72 hours during an 18-day culture. Each condition was normalized to the scramble gRNA, to assess the effect of CRISPR-Cas9 editing on cell proliferation. For statistical comparisons, normality analyses for each group were performed using Shapiro-Wilk test, followed by Levenne test for homogeneity of variances. For parametric group comparisons one-way ANOVA was used, and Kruskall-Wallis test was selected for non-parametric analyses. For multiple-comparisons post-hoc, Tukey correlation was used for samples with homogenous variances, Tamhane's T2 for variance heterogeneity and Fisher's LSD with Bonferroni correction for comparisons among three groups.

Measurement of reactive oxygen species (ROS) by flow cytometry. Cells were harvested and stained with 5 µM CellROX Deep Red Reagent (Thermo Fisher) for 30 minutes at 37°C, following the manufacturer's protocol after 7, 9 and 11 days of gRNAs transduction. Using FACS Canto II (BD Biosciences) and FlowJo flow cytometry analysis software, mean ROS levels (determined as the mean fluorescence intensity in APC) were measured in the BFP^+ subpopulation of viable cells. Cells edited by different gRNAs were then normalized to the same cells transduced with a scramble gRNA. Two independent biological replicates were performed for each condition.

Supplemental References:

Agirre X, Meydan C, Jiang Y, Garate L, Doane AS, Li Z, Verma A, Paiva B, Martín-Subero JI, Elemento O, et al. Long non-coding RNAs discriminate the stages and gene regulatory states of human humoral immune response. Nat Commun. 10, 821 (2019)

Anders S. & Huber W. Differential expression analysis for sequence count data. Genome Biol. 11, R106 (2010).

Brinkman EK, Kousholt AN, Harmsen T, Leemans C, Chen T, Jonkers J & van Steensel B. Easy quantification of template-directed CRISPR/Cas9 editing. Nucleic Acids Res. 46, E58 (2018).

Ecker S, Chen L, Pancaldi V, Bagger FO, Fernández JM, Carrillo de Santa Pau E, Juan D, Mann AL, Watt S, Casale FP, et al. Genome-wide analysis of differential transcriptional and epigenetic variability across human immune cell types. Genome Biol. 18, 18(2017).

Eden E, Navon R, Steinfeld I, Lipson D. & Yakhini Z. GOrilla: a tool for discovery and visualization of enriched GO terms in ranked gene lists. BMC Bioinformatics 10, 48 (2009).

Jaitin DA, Kenigsberg E, Keren-Shaul H, Elefant N, Paul F, Zaretsky I, Mildner A, Cohen N, Jung S, Tanay A, et al. Massively parallel single-cell RNA-seq for marker-free decomposition of tissues into cell types. Science. 343, 776–9 (2014).

Liberzon A, Birger C, Thorvaldsdóttir H, Ghandi M, Mesirov JP, Tamayo P. The Molecular Signatures Database Hallmark Gene Set Collection. Cell Syst. 1, 417–425 (2015).

Lindsay H, Burger A, Biyong B, Felker A, Hess C, Zaugg J, Chiavacci E, Anders C, Jinek M, Mosimann C, et al. CrispRVariants charts the mutation spectrum of genome engineering experiments. Nat. Biotechnol. 34, 701–702 (2016).

Lovén J, Hoke HA, Lin CY, Lau A, Orlando DA, Vakoc CR, Bradner JE, Lee TI, Young RA. Selective inhibition of tumor oncogenes by disruption of super-enhancers. Cell. 153, 320-34. (2013)

McLeay, R. C. & Bailey, T. L. Motif Enrichment Analysis: a unified framework and an evaluation on ChIP data. BMC Bioinformatics 11, 165 (2010).

Merkel A, Fernández-Callejo M, Casals E, Marco-Sola S, Schuyler R, Gut IG, Heath SC. gemBS: high throughput processing for DNA methylation data from bisulfite sequencing. Bioinformatics. bty690(2018).

Pascual M, Alignani D, Vilas-Zornoza A, Delgado JA, Vázquez I, Malumbres R, Rodriguez I, Barriuso R, Calasanz MJ, Paiva B. et al. Use of human pharyngeal and palatine tonsils as a reservoir for the analysis of B-cell ontogeny in 10 paired samples. Clin Otolaryngol. 41, 5(2016).

Serra F, Baù D, Goodstadt M, Castillo D, Filion GJ, Marti-Renom MA. Automatic analysis and 3D-modelling of Hi-C data using TADbit reveals structural features of the fly chromatin colors. PLoS Comput Biol. 13, e1005665 (2017)

Subramanian A, Tamayo P, Mootha VK, Mukherjee S, Ebert BL, Gillette MA, Paulovich A, Pomeroy SL, Golub TR, Lander ES, et al. Gene set enrichment analysis: a knowledge-based approach for interpreting genome-wide expression profiles. Proc. Natl. Acad. Sci. U. S. A. 102, 15545–50 (2005).

Supek F, Bošnjak M, Škunca N & Šmuc T. REVIGO Summarizes and Visualizes Long Lists of Gene Ontology Terms. PLoS One 6, e21800 (2011).

Whyte WA, Orlando DA, Hnisz D, Abraham BJ, Lin CY, Kagey MH, Rahl PB, Lee TI, Young RA. Master transcription factors and mediator establish super-enhancers at key cell identity genes. Cell. 153, 307-19 (2013)

Xie W, Schultz MD, Lister R, Hou Z, Rajagopal N, Ray P, Whitaker JW, Tian S, Hawkins RD, Leung D, et al. Epigenomic analysis of multilineage differentiation of human embryonic stem cells. Cell 153, 5 (2013).

SUPPLEMENTAL FIGURES

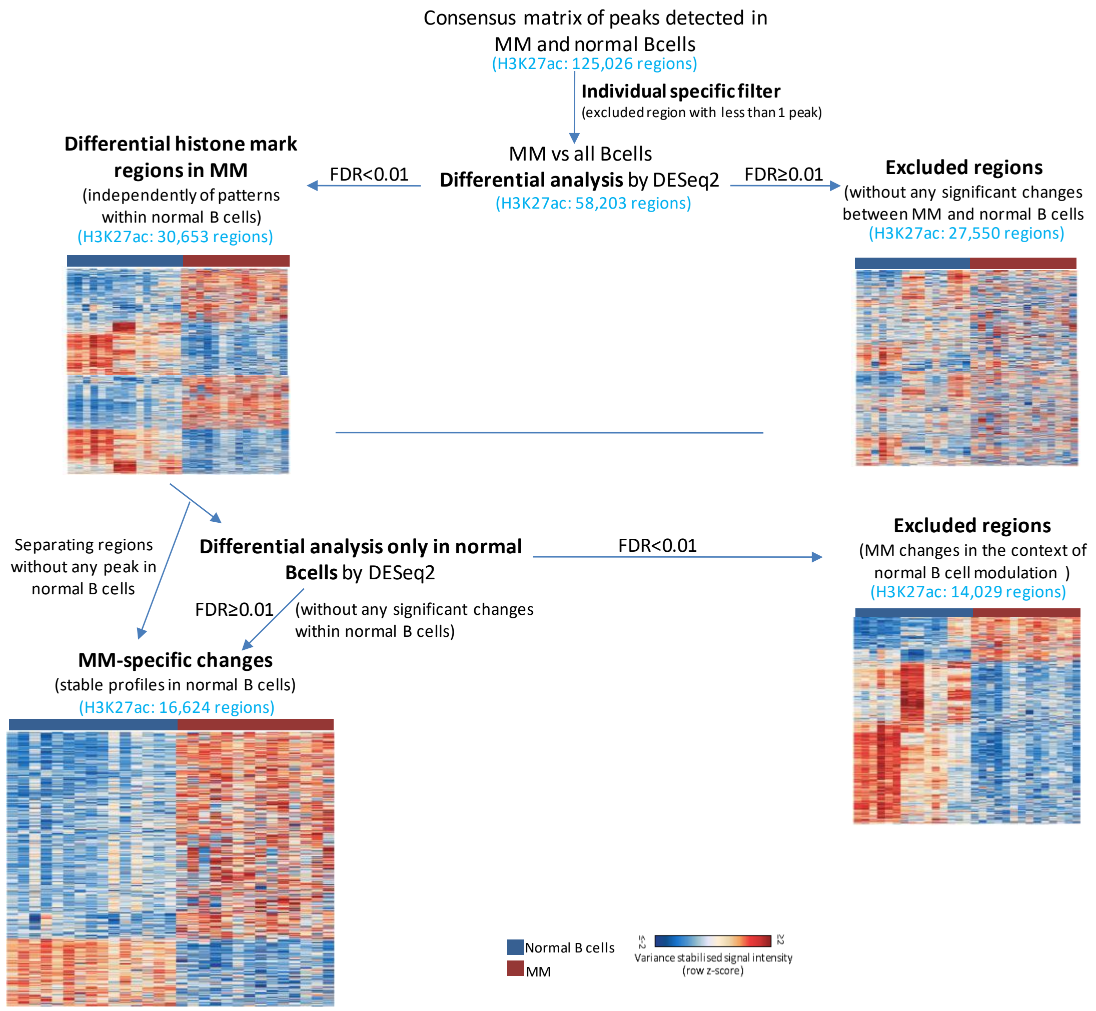

Supplemental Figure S1. Strategy of differential histone marks analysis in MM as compared to normal B cells. As a final step we segregated the regions into those with either dynamic (lower left heatmap) or stable (lower right heatmap) patterns in normal B cell differentiation.

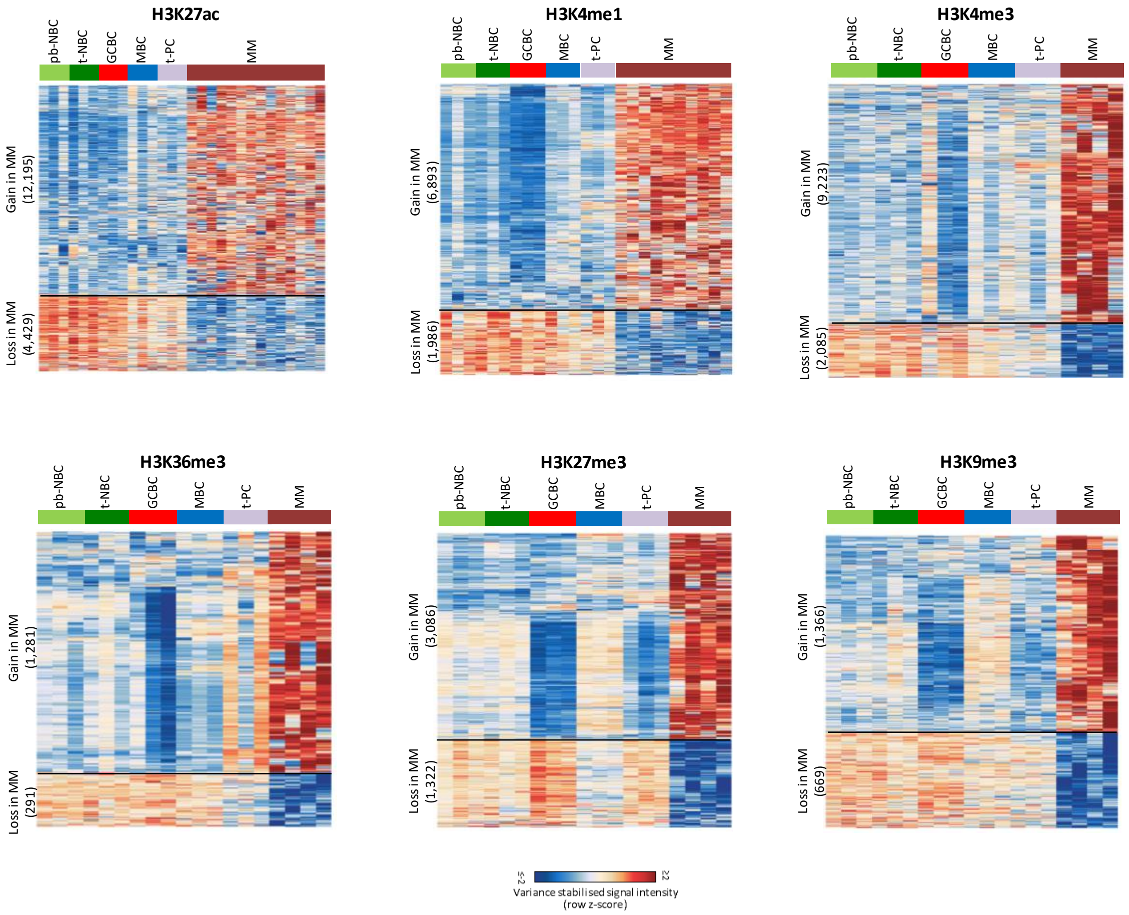

Supplemental Figure S2. Heatmaps representing histone mark changes specifically acquired in MM as compared to normal B cells with stable chromatin profiles throughout cell differentiation. The upper part of each heatmap represent the regions with increase of a particular histone mark in MM (i.e. gain in MM), while the lower part represents the regions with decrease in MM (i.e. loss in MM). Numbers of regions are in brackets. pb-NBC, naive B cells from blood; t-NBC, naive B cells from tonsils; GCBC, germinal centre B cells; MBC, memory B cells; t-PC, plasma cell from tonsils; MM, multiple myeloma.

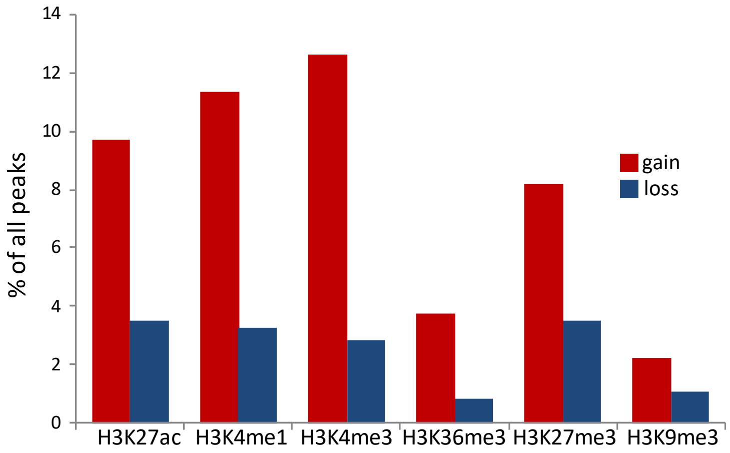

Supplemental Figure S3. Percentages of peaks defined as differential between MM and normal B cells (red bars – gained regions in MM, blue bars – lost regions in MM), relative to all detected peaks per each histone mark

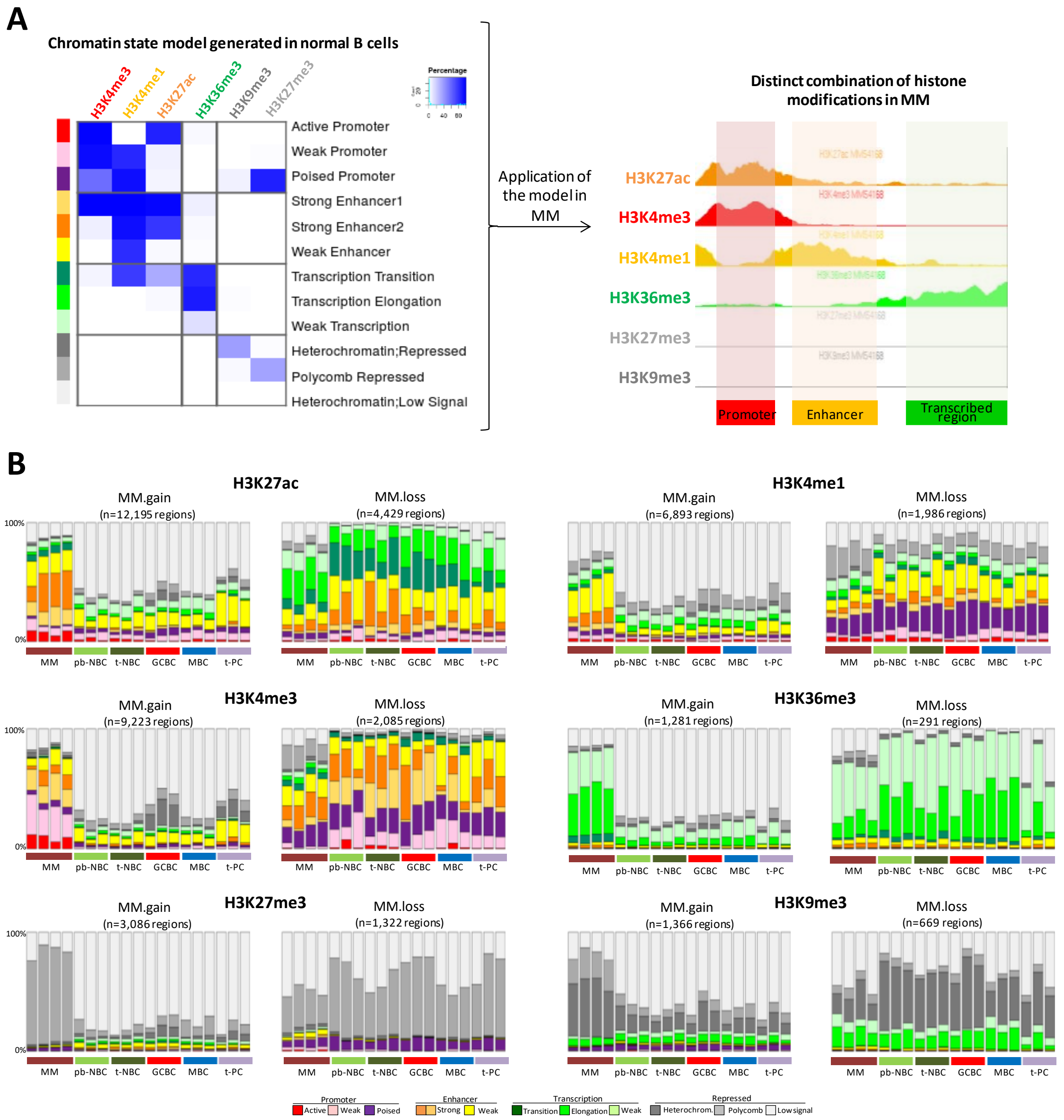

Supplemental Figure S4. A) Schematic representation of chromatin states generation strategy. Left panel shows emissions of the chromatin state model generated in normal B cells (Beekman et al. 2018), where the percentages of regions assigned to a specific chromatin state (rows) that contain a specific histone mark (columns). Right panel shows how this model was applied to our MM data. **B)** Distribution of the different chromatin states in all analyzed samples separately at regions with differential enrichment of histone marks in MM (either gain or loss) as compared to stable chromatin profiles throughout B cell differentiation. pb-NBC, naive B cells from blood; t-NBC, naive B cells from tonsils; GCBC, germinal centre B cells; MBC, memory B cells; t-PC, plasma cell from tonsils; MM, multiple myeloma.

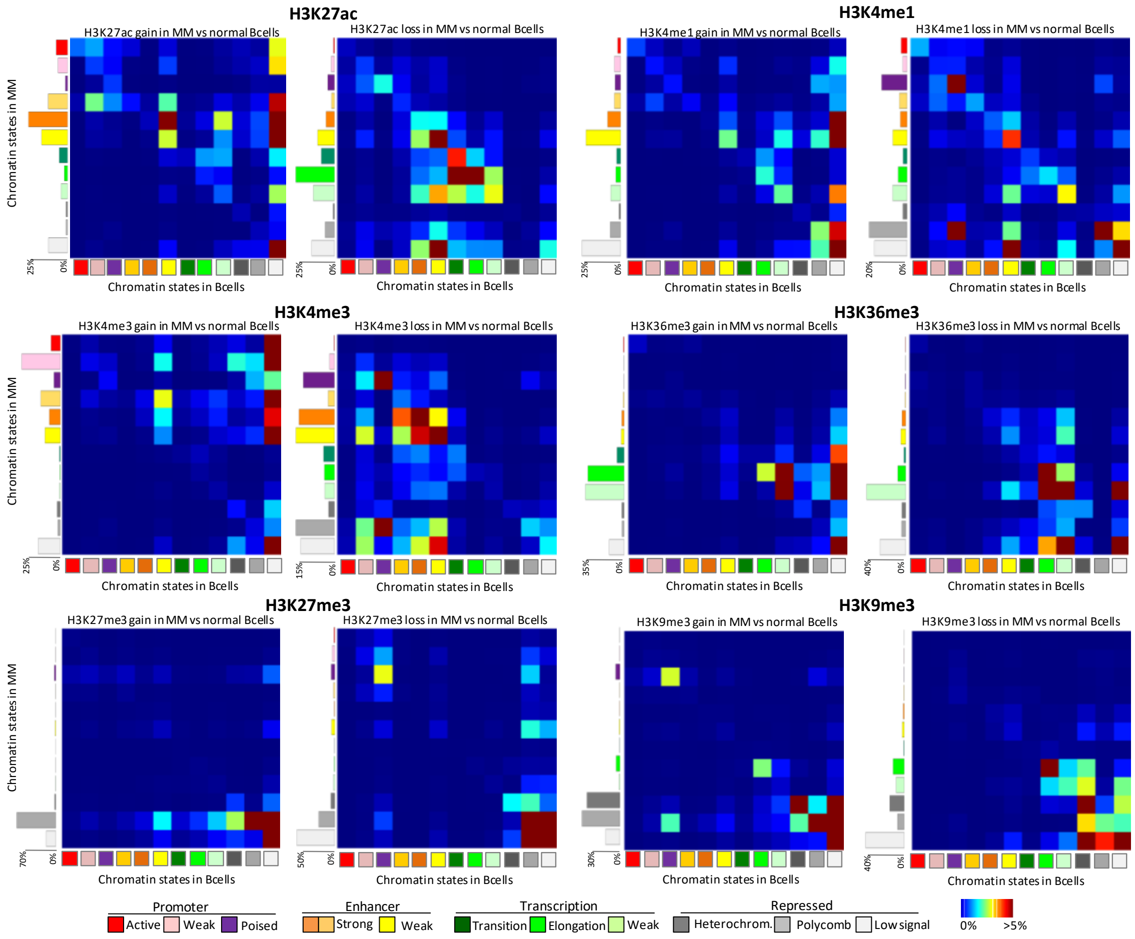

Supplemental Figure S5. Chromatin state transition matrix for regions with differential enrichment of six histone marks in MM (either gain or loss) as compared to normal B cells. Columns represent the chromatin state in normal B cells and rows are chromatin states in MMs that arise from normal B cells. The total matrix represents 100 percent of the differential regions. The percentage of regions associated with each chromatin state in MM patients is indicated in the left side bar chart.

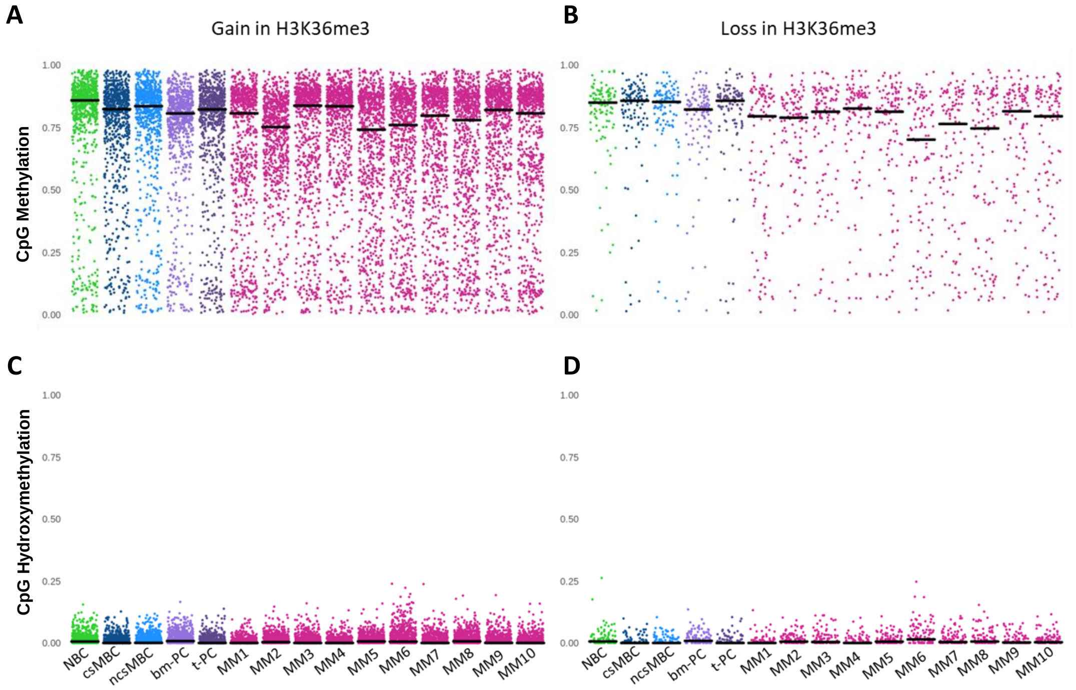

Supplemental Figure S6. Methylation and hydroxymethylation levels of CpGs related to differential H3K36me3 levels. **A) and B)** Methylation levels of CpGs located within regions with gain (A) and loss (B) in H3K36me3 in MM as compared with normal B cells. **C) and D**) Hydroxymethylation levels of CpGs located within regions with gain (C) and loss (D) in H3K36me3 in MM as compared with normal B cells. Black lines correspond to median of all CpG data per sample. NBC, naive B cells; GCBC, germinal center B cells; csMBC, class-switched memory B cells; ncsMBC, non class-switched memory B cells; t-PC, plasma cell from tonsils; bm-PC, plasma cell from bone marrow; MM, multiple myeloma

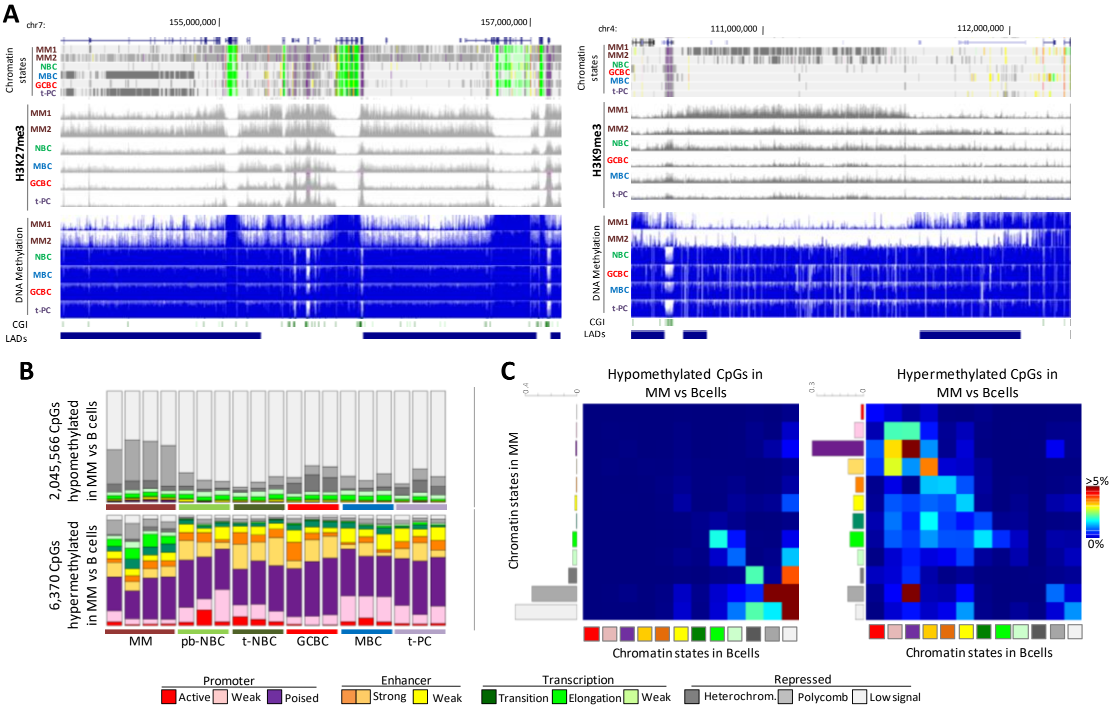

Supplemental Figure S7. A) Schematic representation of large blocks, with increased peaks of H3K27me3 (left) and H3K9me3 (right) in MM, harbouring extensive loss of DNA methylation in neoplastic cells, as compared to normal B cell differentiation. For each panel following tracks, showing different epigenetic features in the very same samples, are represented (from upper to lower): chromatin states segmentation; H3K27me3 (left) or H3K9me3 (right) peaks profile and DNA methylation values (0-1 scale). Additionally tracks showing CpG Island (CGI) and lamin-associated domains (LADs, data from fibroblasts by ENCODE) are added. We detected 26,537 of these large blocks of DNA hypomethylated regions (DNA methylation valleys, range size 5kb-40kb), which as a whole span over 1.8% of the whole genome and contains 3% of all CpGs detected by WGBS. **B)** Distribution of the different chromatin states in all analyzed samples separately at CpG sites hypomethylated or hypermethylated in MM as compared to normal B cell differentiation. **C)** Chromatin state transition matrix for CpG sites hypomethylated or hypermethylated in MM as compared to normal B cell differentiation. Columns represent the chromatin state in normal B cells and rows are chromatin states in MMs that arise from normal B cells. The total matrix represents 100 percent of the differential regions. The percentage of regions associated with each chromatin state in MM patients is indicated in the left side bar chart. pb-NBC, naive B cells from blood; t-NBC, naive B cells from tonsils; GCBC, germinal centre B cells; MBC, memory B cells; t-PC, plasma cell from tonsils; MM, multiple myeloma.

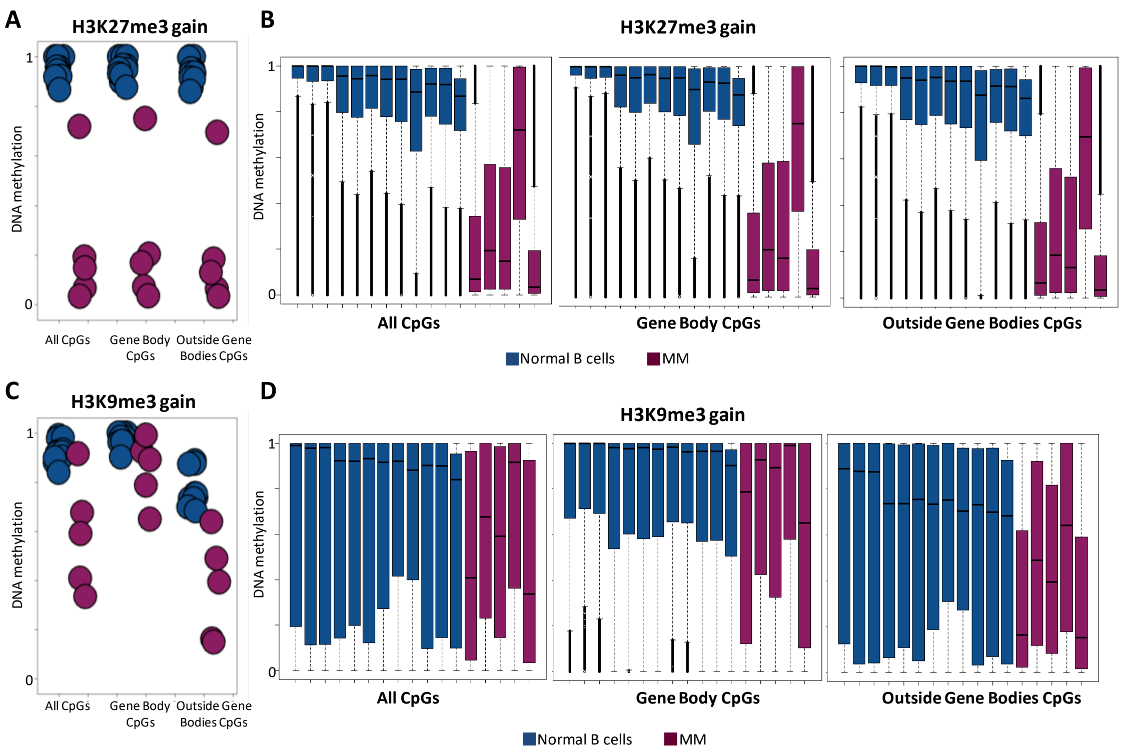

Supplemental Figure S8. DNA Methylation analysis of gene body CpGs within inactive chromatin histone marks. **A) and C)** Scatter plots representing median methylation levels of all CpGs (left), CpGs within gene bodies (middle) and CpGs outside gene body (right) in normal B cells (blue) and MM (purple), within regions with either H3K27me3 (A) or H3K9me3 (C) gain. **B) and D)** Boxplot representing methylation of all CpGs (left panel), CpGs within gene bodies (middle panel) and CpGs outside gene body (right panel) in normal B cells (blue) and MM (purple), within regions with either H3K27me3 (B) or H3K9me3 (D) gain.

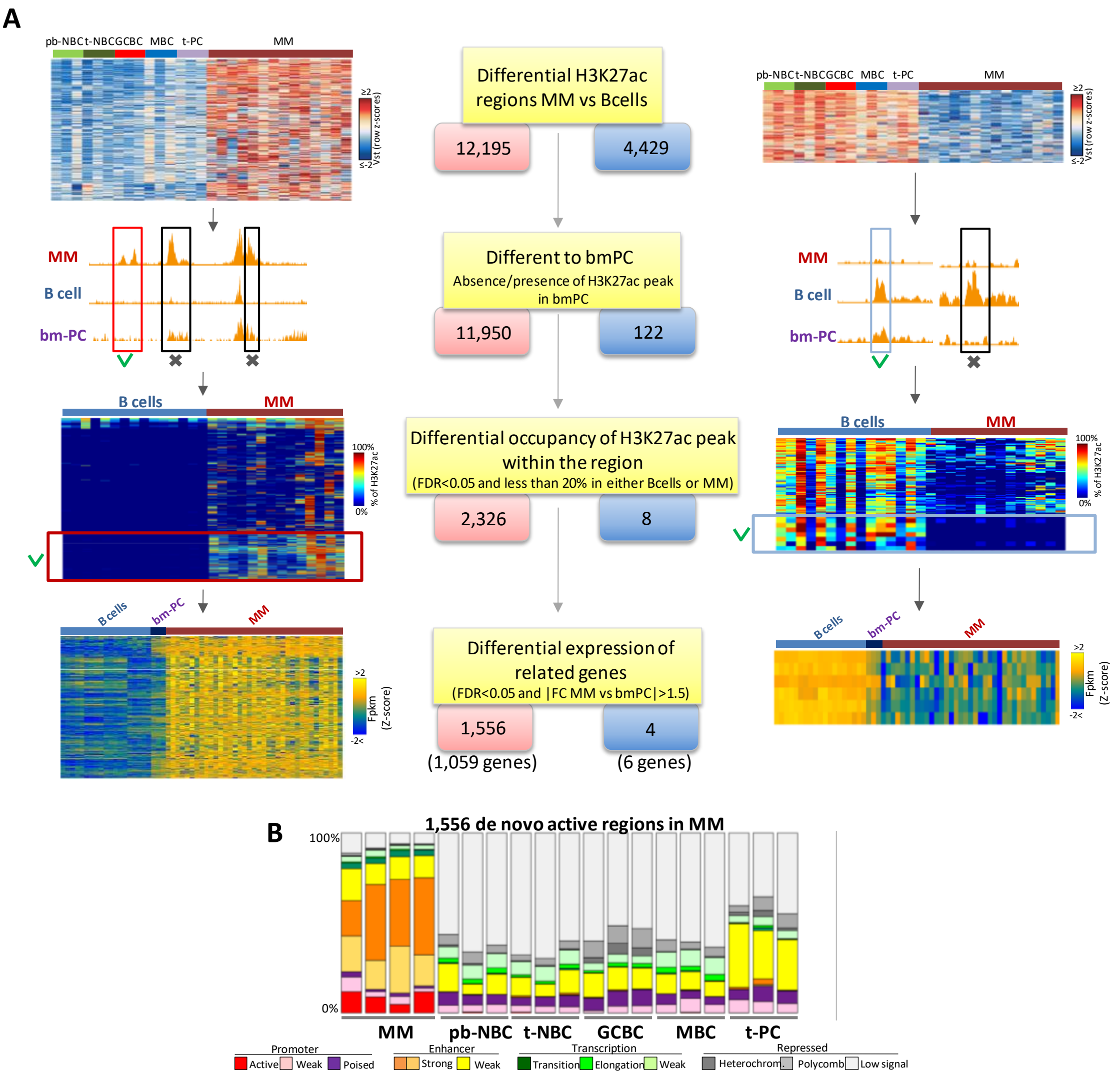

Supplemental Figure S9. A) Schematic representation of strategy to determine *de novo* active regions in MM, that is these without any H3K27ac peak across normal B cell differentiation including bm-PCs (i.e. inactive in normal B cells), while gaining this histone mark only in MM (numbers in red boxes and left panels). An opposite analysis in order to detect *de novo* inactive regions in MM was performed in parallel (numbers in blue boxes and right panels). For a detailed explanation of each step please refer to Methods section. Graphical representation of regions selected in each step is shown on left (for regions *de novo* active in MM) and right (for regions *de novo* inactive in MM) from the schematic workflow. **B)** Distribution of the different chromatin states in all analyzed samples within the 1,556 *de novo* active regions in MM. pb-NBC, naive B cells from blood; t-NBC, naive B cells from tonsils; GCBC, germinal centre B cells; MBC, memory B cells; t-PC, plasma cell from tonsils; bm-PC, plasma cell from bone marrow; MM, multiple myeloma.

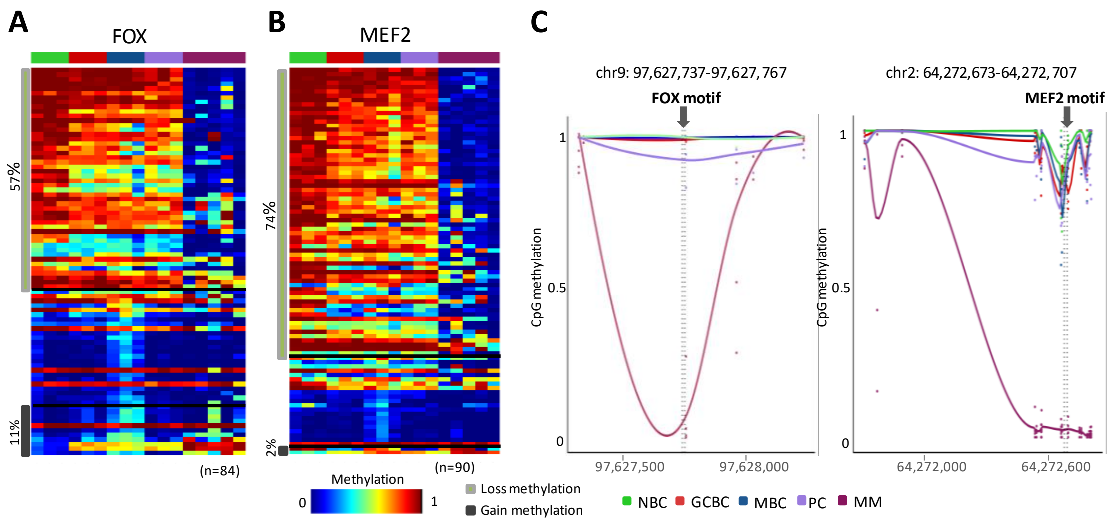

Supplemental Figure S10. DNA Methylation levels associated with regions containing FOX and MEF2 motifs within *de novo* active regulatory elements. **A)** and **B)** Heatmaps representing methylation levels of all CpGs identified in the proximity of FOX (**A**) and MEF2 (**B**) motifs, in different subpopulations of normal B cells and MM samples. Light grey bar marks the CpGs hypomethylated in MM; dark grey bar marks the CpGs hypermethylated in MM. **C)** Examples of DNA methylation profiles in the regions containing FOX and MEF2 motifs (left and right panel, respectively). Grey arrows and vertical dashed lines show TF motifs.

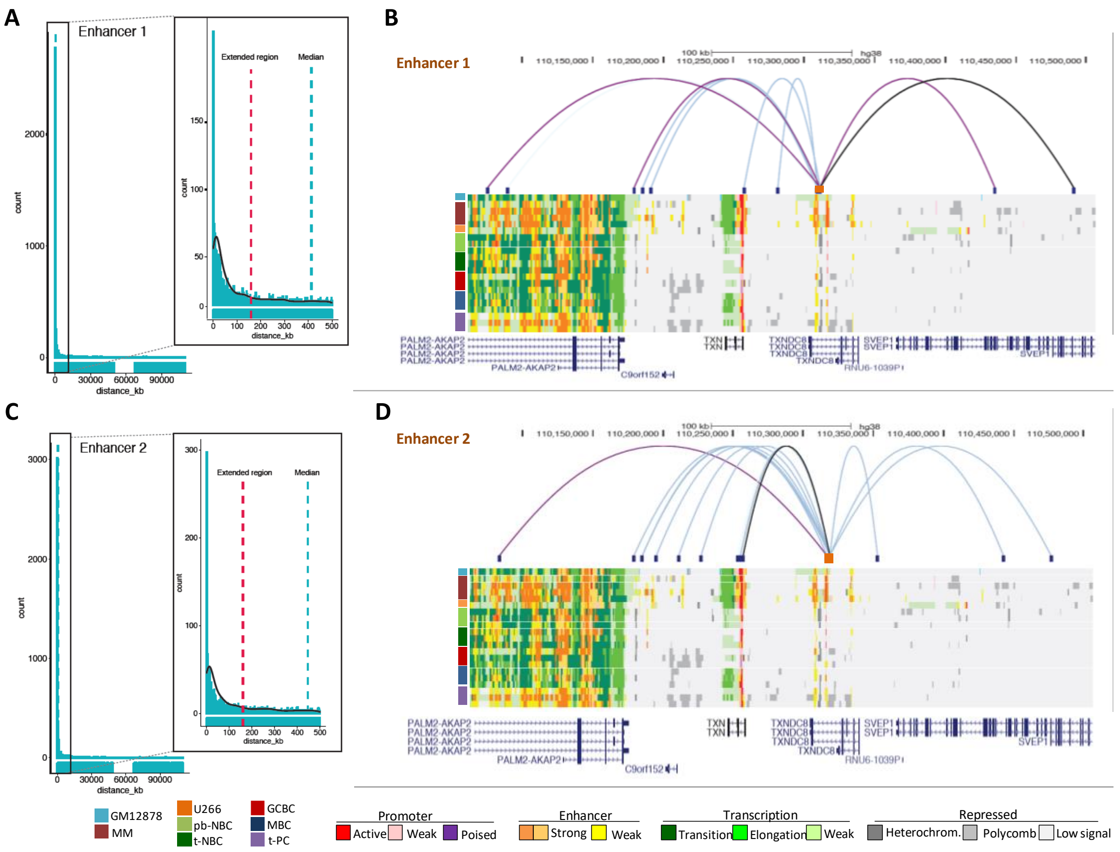

Supplemental Figure S11. 3D interactions of regulatory region associated with *TXN* gene. This analysis was done separately for two regulatory regions, located around 80kb from *TXN* gene, identified as enhancer 1 (chr9: 110,307,163-110,310,535) and enhancer 2 (chr9: 110,314,762-110,318,424) locus. **A)** and **C)** Density plots representing interactions of enhancer 1 (**A**) or enhancer 2 (**C**) throughout whole chromosome 9 as a function of distance. In the zoomed in panel, we could observe that the vast majority of the interactions occur at a distance shorter than 100kb, median size of interaction is marked with blue dashed line (around 400kb), while dark pink dashed line represent the size of the extended region of our enhancers of interest for which more detailed analysis of the interactions was performed. **B)** and **D)** Significant interactions of enhancer 1 (**B**) and 2 (**D**) (marked with orange squares) within the extended region (160kb upstream and downstream of our region of interest). Darker color of the lines corresponds to higher strength of the interactions. Besides clear 3D interactions of both enhancer loci with the *TXN* promoter region, some interactions with intergenic, heterochromatic regions are observed (possibly important structurally). The observed interactions with intronic regions of *PALM2-AKAP2* gene did not seem to have any apparent regulatory role, as we did not observe any expression difference of this gene in MM and normal B cells (data not shown). Below 3D interactions graphs, chromatin state tracks of GM12878 cell lines, as well as MMs and B cells are shown.

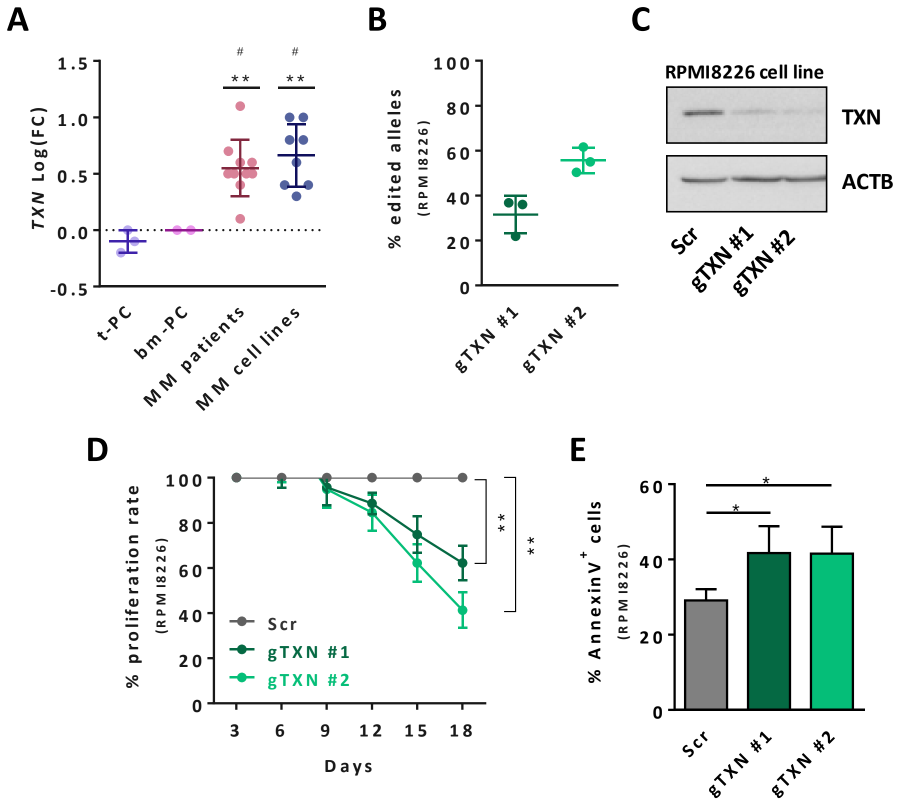

Supplemental Figure S12. A) *TXN* overexpression validation in an independent cohort of MM patients and cell lines versus t-PC (* p <0.05, ** p < 0.01, *** p < 0.001, ns: not significant) and bm-PC (# p <0.05, ## p < 0.01, ### p < 0.001, ns: not significant) as determined by RT-qPCR. **B)** Estimation of the allelic cell population percentage exhibiting indel events in the targeted site of the RPMI8226 cell line, analyzed by Tracking of Indels by DEcomposition (TIDE) web tool. **C)** Validation of TXN knockout by western blot analysis in the RPMI8226 cell line. **D)** Cell proliferation rates of scramble cells (Scr) and cells harboring two different gRNAs in RPMI8226 cell line, as determined by flow cytometry analysis. **E)** Effect of TXN knockout on cell apoptosis in the RPMI8226 cell line, as determined by Annexin V flow cytometry analysis. * p<0.05, ** p<0.01, *** p<0.001, ns: not significant.

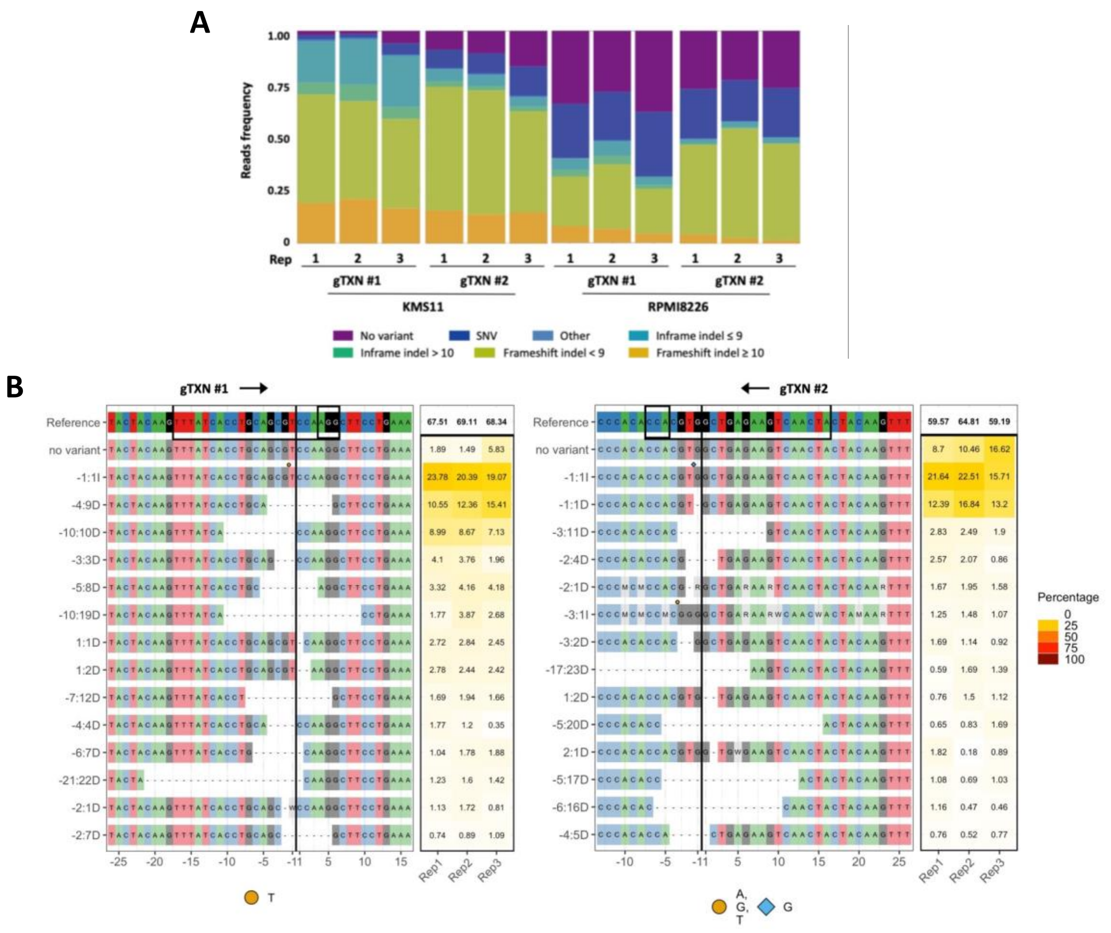

Supplemental Figure S13. A) Characterization of the variants introduced in *TXN* coding sequence analyzed by NGS, grouped according to their type, size, and frameshift potential. Each bar represents a biological replicate (Rep). **B)** Summary of most frequent CRISPR-Cas9 variant types introduced in *TXN* gene sequence. Characterization of variant types, locations and frequency in a pool of KMS-11 MM cells after CRISPR-Cas9-mediated *TXN* gene editing for gTXN #1 (**left**) and gTXN #2 (**right**). The left panel shows an alignment of the consensus sequence for each variant combination to the reference sequence (top), relative to the gRNA target sequence (squared, including the PAM -NGG or -NCC). Deleted nucleotides are shown as dashes. Inserted nucleotides are shown on top of the alignments, indicated by different symbols (key at bottom of figure). The right panel shows a heat map of the frequency of the variants in the cell pool across the different replicates. As a header, the column sum of variants frequencies shown in the plot for each replicate.

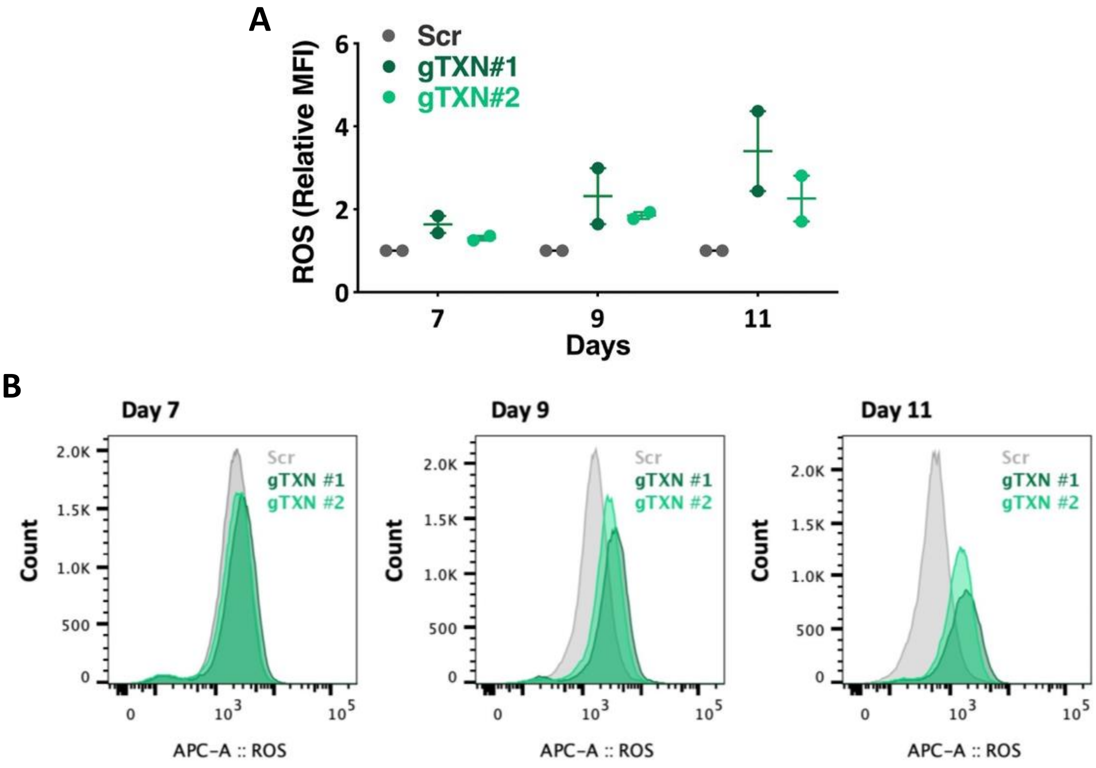

Supplemental Figure S14. Reactive Oxygen Species (ROS) levels after CRISPR-Cas9-mediated *TXN* gene or enhancer editing. **A)** Quantification of ROS levels after CRISPR-Cas9-mediated TXN gene edition in KMS-11 cell line. Values are presented as relative Mean Fluorescence Intensities (MFI) normalized to the same cells infected with a scramble gRNA (Scr). Values of two independent experiments and the median ± 95% confidence intervals are shown (median fold change increase of 3.4 and 2.26 for each gRNA, respectively). **B)** Flow cytometry histograms showing changes in ROS levels after CRISPR-Cas9-mediated *TXN* gene editing, 7, 9 and 11 days after gRNA transduction in KMS-11 cell line.

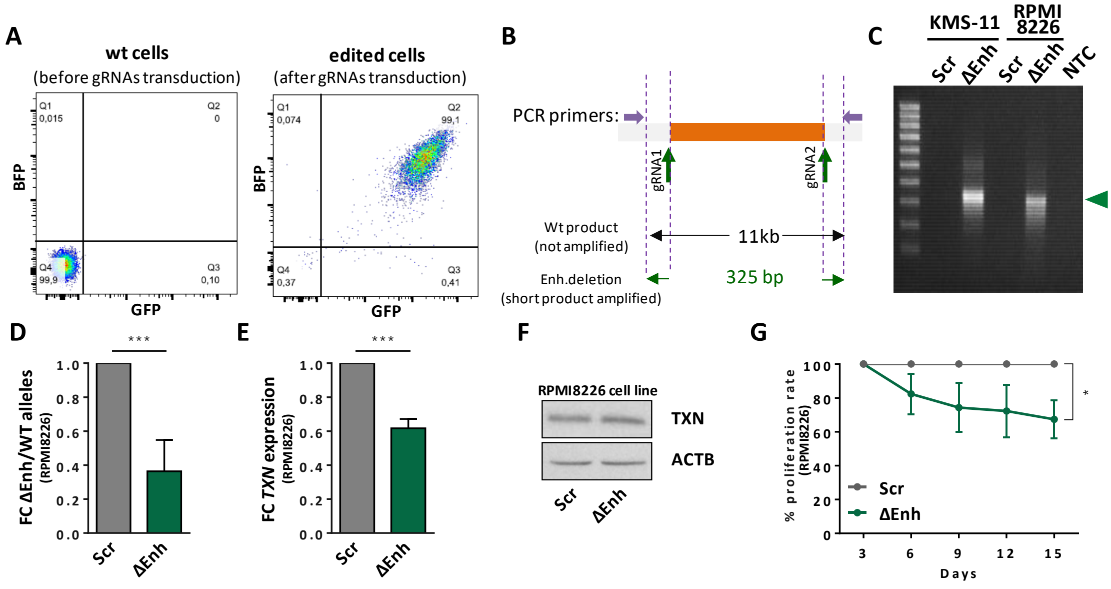

Supplemental Figure S15. A) Fraction of double positive BFP+/GFP+ cells analyzed by flow cytometry prior to (left) and after paired gRNAs transduction (right). Upper right quadrant (Q2) shows double BFP+ / GFP+ cells. The cell number proportion of each subpopulation is shown in each quadrant. **B)** Primer design strategy for qualitative analysis of *TXN* enhancer deletion. External primers flanking the enhancer target region (11 kb) were designed to detect the presence of smaller PCR products (325 bp) in edited alleles by gel electrophoresis. **C)** Qualitative analysis of *TXN* enhancer deletion by electrophoresis of genomic DNA PCR products. Wild type genomic DNA (scramble cells, Scr) and water (NTC) templates are used as negative controls. The green arrowhead indicates the size of PCR products expected from the deleted allele. **D)** Quantification of *TXN* enhancer deletion in the RPMI8226 cell line by genomic DNA qPCR normalized to a distal non-targeted genomic region, represented as fold change of deleted enhancer (ΔEnh) versus wild type (WT) alleles. **E)** *TXN* mRNA expression levels determined by RT-qPCR in the RPMI8226 cell line. **F)** TXN protein levels determined by western blot in the RPMI8226 cell line. **G)** Cell proliferation rates of Scr cells and cells harboring the enhancer deletion in RPMI8226 cell line, as determined by flow cytometry analysis. * $p<0.05$, ** $p<0.01$, *** $p<0.001$, ns: not significant.

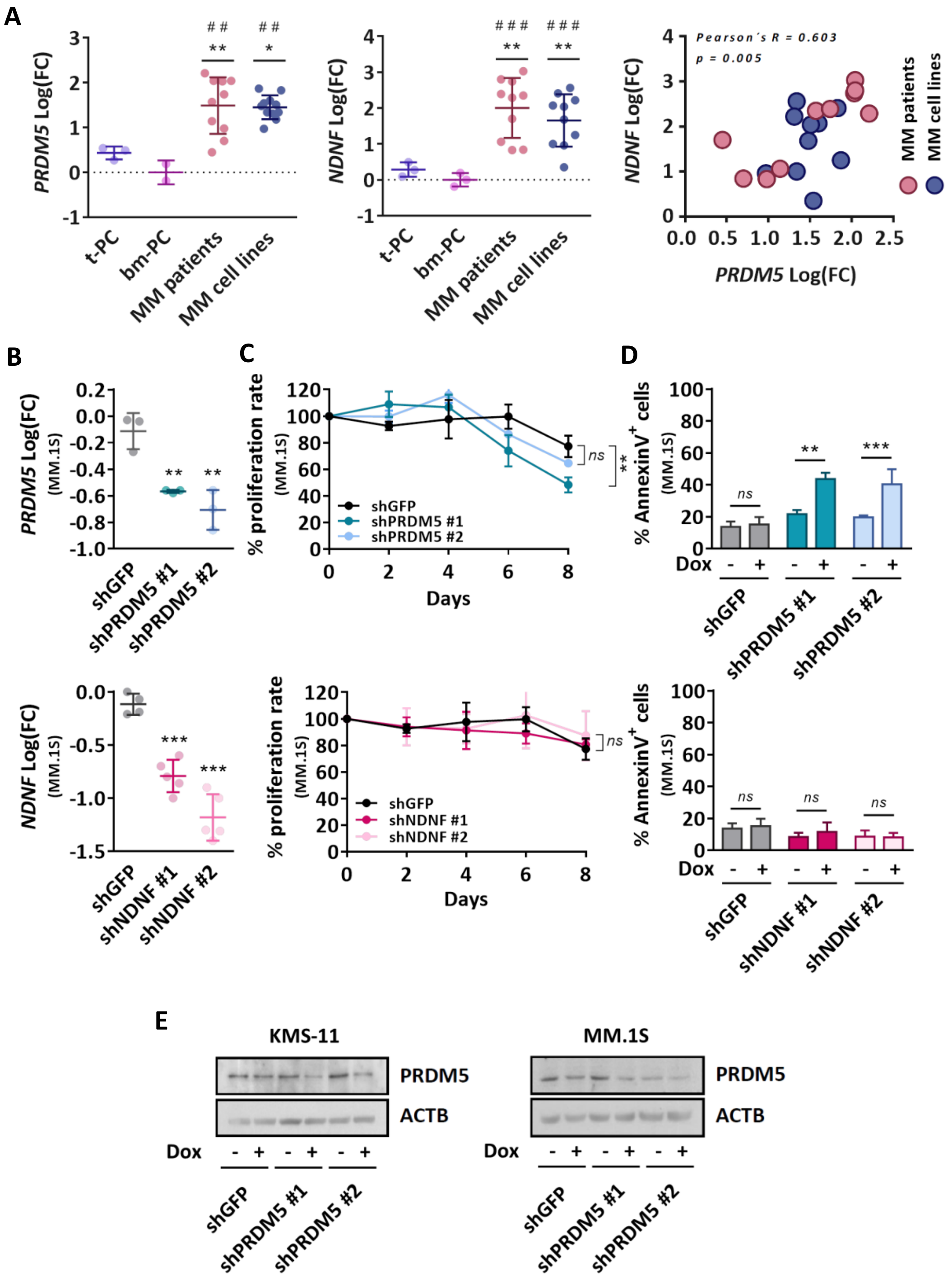

Supplemental Figure S16. A) *PRDM5* and *NDNF* overexpression validation in an independent cohort of MM patients and cell lines versus t-PC (* p<0.05, ** p<0.01, *** p<0.001, ns: not significant) and bm-PC (# p<0.05, ## p<0.01, ### p<0.001, ns: not significant) as determined by RT-qPCR, and correlation of expression levels of both transcripts (right panel). **B)** Validation of *PRDM5* (top) and *NDNF* (bottom) gene knockdown after shRNA expression determined by RT-qPCR in the MM.1S cell line. Expression values are normalized to the same condition prior to addition of doxycycline. Statistical analysis compares the effect of each shRNA versus the scramble shRNA (shGFP). **C)** Relative cell proliferation rate (%) of the MM.1S cell line normalized to the same condition prior to addition of doxycycline. Upper panel present the results for

PRDM5 knockdown cells and lower panel for *NDNF* knockdown cells. Statistical analysis compares the effect of each shRNA versus the scramble shRNA (shGFP). **D)** Effect of *PRDM5* (upper panel) and *NDNF* (lower panel) knockdown in cell apoptosis, as determined by Annexin V flow cytometry analysis in the MM.1S cell line. **E)** Reduction of PRDM5 protein levels after shRNA induction determined by western blot in the KMS-11 and MM.1S cell lines. Dox: Doxycycline. * p<0.05, ** p<0.01, *** p<0.001, ns: not significant.

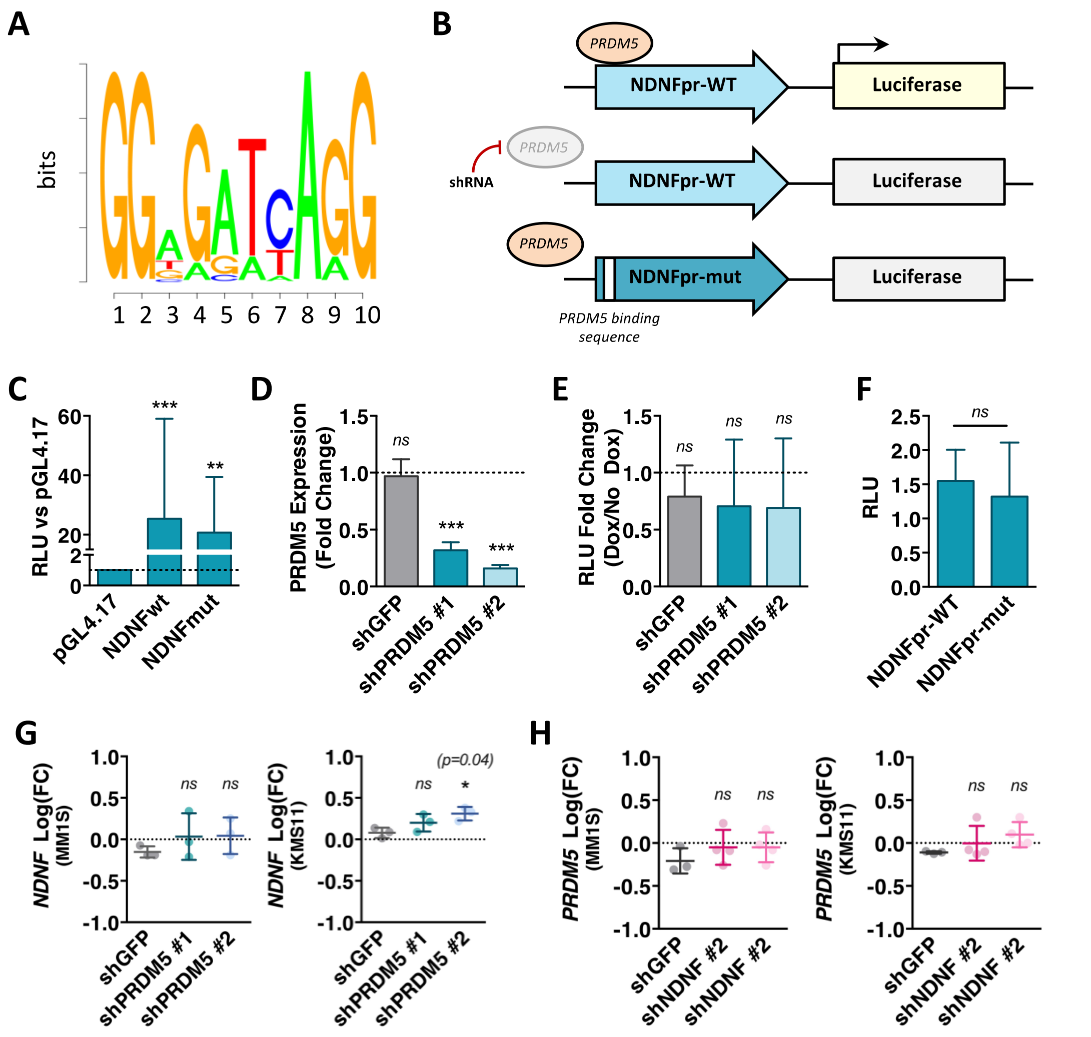

Supplemental Figure S17. Influence of PRDM5 in NDNF expression. **A**) Sequence logo representation of PRDM5 consensus binding sequence. Mapping of this motif to *NDNF* promoter region (-1.5 kb from TSS) and gene body, has identified one putative PRDM5 binding site (GGAGAGCCGG) in *NDNF* promoter region **B**) Schematic representation of the luciferase reporter assay designed to assess the potential role of PRDM5 transcription factor in NDNF overexpression. Luciferase gene expression is driven by *NDNF* promoter (NDNFpr-WT). If PRDM5 was regulating the NDNF expression, transcription factor knockdown (mediated by shRNAs) or deletion of its putative binding sequence (NDNFpr-mut, deleted region is represented as a white gap) would impair the transcription of luciferase reporter gene. **C**) Relative luciferase/renilla units (RLU) for empty vector (pGL4.17), *NDNF* wild type promoter (NDNFpr-WT) and *NDNF* promoter harboring PRDM5 binding site deletion (NDNFpr-mut). Values are normalized to the pGL4.17 empty vector. **D**) PRDM5 expression levels in HEK293T cells after shRNA expression determined by RT-qPCR, using GUSB for gene normalization. Expression values are normalized to the same condition prior to addition of doxycycline. **E**) Lack of modulation of luciferase/renilla values (RLU Fold Change) after shRNA mediated PRDM5 knockdown in HEK293T cells. Values are normalized to the same condition prior to addition of doxycycline. **F**) Lack of luciferase reporter activity (RLU) for *NDNF* wild type promoter (NDNFpr-WT) and *NDNF* promoter harboring PRDM5 binding site deletion (NDNFpr-mut) in HEK293T cells. **G**) *NDNF* expression levels in MM cell lines after PRDM5 silencing determined by RT-qPCR, using *GUSB* for gene normalization. Expression

values are normalized to the same condition prior to addition of doxycycline. **H**) *PRDM5* expression levels in MM cell lines after NDNF silencing determined by RT-qPCR, using *GUSB* for gene normalization. Expression values are normalized to the same condition prior to addition of doxycycline. * $p < 0.05$, ** $p < 0.01$, *** $p < 0.001$, ns: not significant.

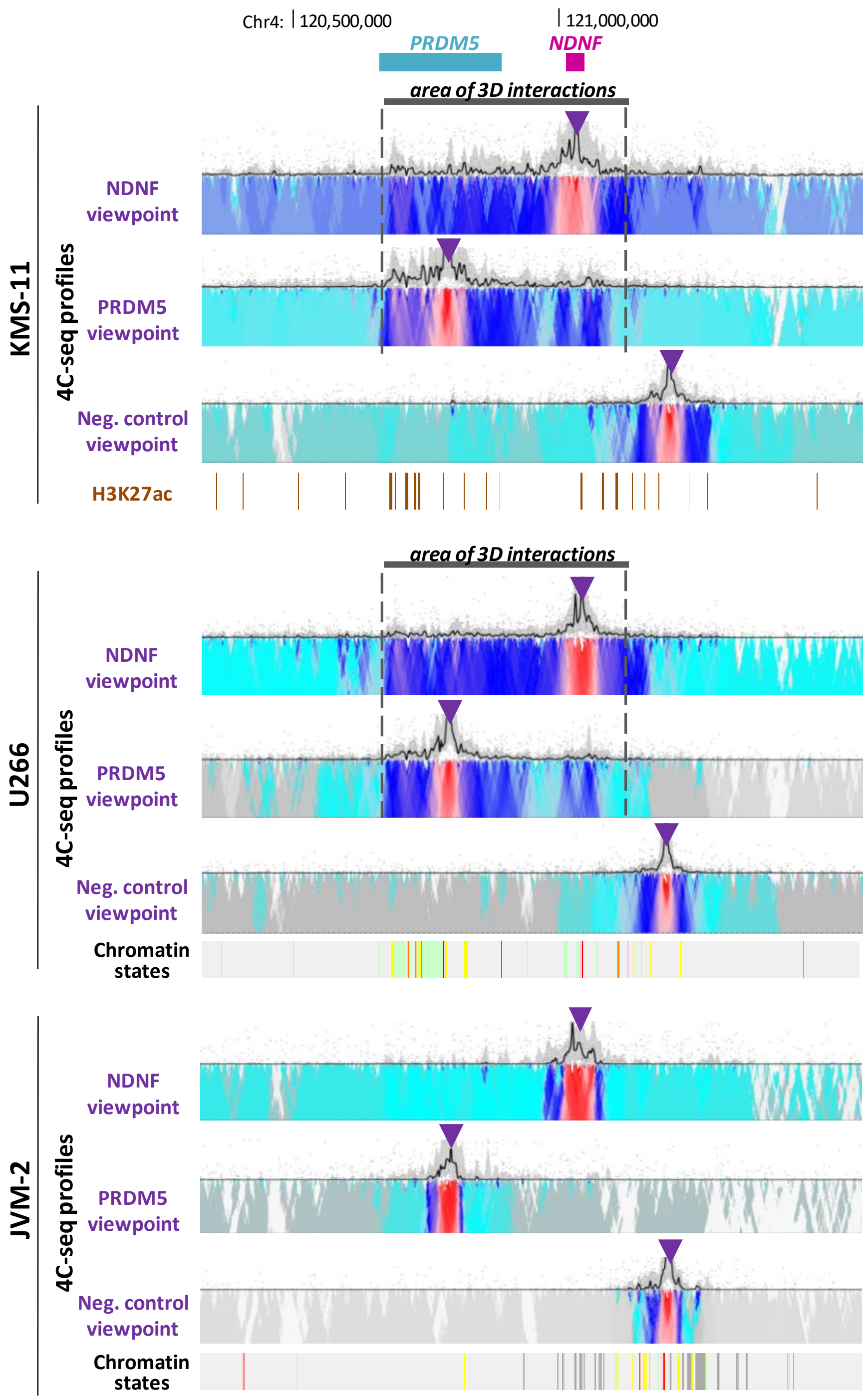

Supplemental Figure S18. 3D interaction within *PRDM5-NDNF* locus, as measured by 4C-seq in three different cell lines, from top to bottom: KMS-11, U266 and JVM-2 (used as negative control). Normalized levels of chromatin interaction frequencies from the indicated viewpoint (purple arrowhead) between *PRDM5* and *NDNF* gene loci as analyzed by 4C-seq. In each cell line, three different viewpoints were analyzed, i.e. viewpoint located within *NDNF* promoter (the upper panels), viewpoint within *PRDM5* gene (middle panels) and viewpoint near *TNIP3*, as a negative control (the lower panels). Lowest contact frequencies are indicated in turquoise, and highest frequencies in red. *PRDM5*, *NDNF* and *TNIP3* location together with chromatin state tracks for JVM-2 and U266 samples and H3K27ac tracks for KMS-11 are shown above 4C-seq track

SUPPLEMENTAL TABLES

List of separately provided tables:

Supplemental Table S1. Experimental design. MM and normal B cell cases used for the study

Supplemental Table S2. Characterization of Multiple Myeloma (MM) patient samples.

Supplemental Table S3. List of *de novo* activated regions in MM with associated target genes.

Supplemental Table S4. List of super-enhancers detected in the 1,556 *de novo* active chromatin regions.

Supplemental Table S5. TFBS enrichment analysis by MEME. TF motifs analysis in 806 sites with increased chromatin accessibility in MM, within the 1,556 regions *de novo* active in MM.

Supplemental Table S6. Expression of all members of IRF, FOX and MEF2 transcription factor families. FPKM values from RNA-Seq of additional MM and normal B cell series are shown.

Supplemental Table S7. Functional categories analyses associated with 1,059 genes related to *de novo* activated regions in MM. Common terms between all the methods, belonging to one of three main categories: 1) related to ossification/osteoblast differentiation, 2) related to main signaling pathways or 3) related to oxidative stress or stress response, are highlighted in different color

Supplemental Table S8. Genes included in co-regulated chromatin regions. Gene pair selected for downstream analysis is shaded.

Supplemental Table S9. Primers and target sequences used in this study
